# Non-canonical regulation of SPL transcription factors by a human OTUB1-like deubiquitinase defines a new plant type rice associated with higher grain yield

**DOI:** 10.1038/cr.2017.98

**Published:** 2017-08-04

**Authors:** Shuansuo Wang, Kun Wu, Qian Qian, Qian Liu, Qi Li, Yajun Pan, Yafeng Ye, Xueying Liu, Jing Wang, Jianqing Zhang, Shan Li, Yuejin Wu, Xiangdong Fu

**Affiliations:** 1 The State Key Laboratory of Plant Cell and Chromosome Engineering, Institute of Genetics and Developmental Biology, Chinese Academy of Sciences, National Centre for Plant Gene Research, Beijing 100101, China; 2 Key laboratory of high magnetic field and Ion beam physical biology, Hefei Institutes of Physical Science, Chinese Academy of Sciences, Hefei, Anhui 230031, China; 3 School of Life Sciences, University of Science and Technology of China, Hefei, Anhui 230000, China; 4 The State Key Laboratory of Rice Biology, China National Rice Research Institute, Hangzhou, Zhejiang 310006, China; 5 College of Life Sciences, University of Chinese Academy of Sciences, Beijing 100049, China

**Keywords:** new plant type rice, OsOTUB1, deubiquitination, OsSPL14, grain yield

## Abstract

Achieving increased grain productivity has long been the overriding focus of cereal breeding programs. The ideotype approach has been used to improve rice yield potential at the International Rice Research Institute and in China. However, the genetic basis of yield-related traits in rice remains unclear. Here, we show that a major quantitative trait locus, *qNPT1*, acts through the determination of a 'new plant type' (NPT) architecture characterized by fewer tillers, sturdier culms and larger panicles, and it encodes a deubiquitinating enzyme with homology to human OTUB1. Downregulation of *OsOTUB1* enhances meristematic activity, resulting in reduced tiller number, increased grain number, enhanced grain weight and a consequent increase in grain yield in rice. Unlike human OTUB1, OsOTUB1 can cleave both K48- and K63-linked polyubiquitin. OsOTUB1 interacts with the E2 ubiquitin-conjugating protein OsUBC13 and the squamosa promoter-binding protein-like transcription factor OsSPL14. OsOTUB1 and OsSPL14 share common target genes, and their physical interaction limits K63-linked ubiquitination (K63Ub) of OsSPL14, which in turn promotes K48Ub-dependent proteasomal degradation of OsSPL14. Conversely, loss-of-function of *OsOTUB1* is correlated with the accumulation of high levels of OsSPL14, resulting in the NPT architecture. We also demonstrated that pyramiding of high-yielding *npt1* and *dep1-1* alleles provides a new strategy for increasing rice yield potential above what is currently achievable.

## Introduction

Rice feeds more than half the world's population, and improving the productivity of this grain is necessary for food security. Despite the major improvement in grain yield delivered by the exploitation of semi-dwarfism and heterosis^1,2,3^, increasing rice yield potential over that of existing elite cultivars is a major challenge for breeders^4^. In an effort to overcome the yield ceiling of current rice varieties, the ideotype approach has been proposed and used in breeding programmes to improve rice yield potential^4,5,6,7^. Since the early 1990s, a number of 'new plant type' (NPT) rice varieties have been bred at the International Rice Research Institute (IRRI). The architecture of these plants differs from that of conventional varieties: they produce larger panicles and stronger culms and exhibit fewer sterile tillers. Although several NPT rice strains have been commercially released^6,7^, the genetic basis of their phenotypes has been explained only at the level of quantitative trait loci (QTLs)^8^.

The grain yield of rice plants is multiplicatively determined by three main components (the number of panicles per plant, the number of grains per panicle and mean grain weight), all of which are controlled by a number of QTLs derived from natural variation. Several alleles that regulate these complex traits have been shown to improve the yield potential of rice: *Gn1a*, *DEP1* and *GNP1* regulate grain number^5,9,10^; *GS3*, *TGW6* and *OsSPL16* regulate grain size^11,12,13,14^; and *Ghd7*, *NAL1* and *OsSPL14* regulate optimal plant architecture^15,16,17,18^. However, the molecular mechanisms underlying how other QTLs regulate grain yield remain largely unknown. Thus, the identification of alleles improving grain productivity would facilitate the breeding of new high-yield rice varieties and may also be applicable to other crops.

Modification of DELLA proteins with ubiquitin was crucial for the success of the Green Revolution^19^. Ubiquitination usually occurs at lysine (K) side chains of a target protein through a process involving the coordinated action of an E1-ubiquitin-activating enzyme, an E2-ubiquitin-conjugating enzyme and an E3-ubiquitin ligase enzyme. The ubiquitin molecules within polyubiquitin chains can be linked through different lysine residues, but K48- and K63-linked polyubiquitinations are the most frequent modifications detected thus far. Ubiquitination is a reversible process that involves several deubiquitinating enzymes, which dissociate ubiquitin moieties from their protein substrates. Previous studies have shown that K63-linked ubiquitination and deubiquitination are critical post-translational regulatory mechanisms for the recruitment of repair proteins to sites of DNA double-strand breaks^20,21^. Although human ovarian tumor domain-containing ubiquitin aldehyde-binding protein 1 (OTUB1) was previously identified as a K48 linkage-specific deubiquitinating enzyme that positively regulates p53 stability^22^, OTUB1 strongly suppresses E2-ubiquitin-conjugating enzyme UBC13-dependent K63-linked ubiquitination^20,21^. Recent structural and biochemical analyses have elucidated the mechanism underlying the inhibition of UBC13 and other E2 enzymes by OTUB1^21^, but the molecular mechanisms driving the interplay between K48- and K63-linked deubiquitination by OTUB1 have not been completely explained.

Here, we show that a gene encoding a human OTUB1-like deubiquitinating enzyme functions as a key QTL responsible for OsSPL14-mediated control of the NPT architecture and is associated with reduced tiller number, increased lodging resistance and higher grain yield in rice.

## Results

### Identification of *qNPT*1, a major QTL associated with NPT rice

**Figure 1:**
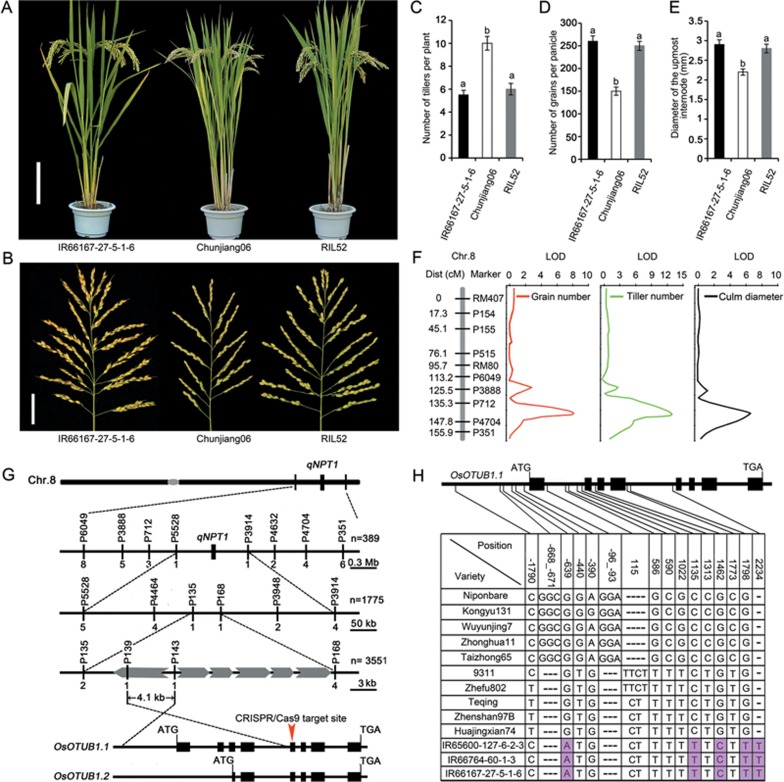
Positional cloning of *qNPT1*. **(A)** Appearance of a mature plant. RIL52 was selected from a Chunjiang06 (CJ06) × IR66167-27-5-1-6 cross. Scale bar, 20 cm. **(B)** Panicle morphology. Scale bar, 5 cm. **(C**-**E)** Comparison of RIL52 with its parents with respect to **(C)** grain number, **(D)** tiller number and **(E)** culm diameter. Data are shown as the mean ± SEM (*n* = 30). The presence of the same lowercase letter denotes a non-significant difference between means (*P* < 0.05). **(F)** QTL mapping for grain number, tiller number and culm diameter. **(G)** Positional cloning of *qNPT1.* The locus was mapped to a ∼ 4.1 Kbp genomic region flanked by P139 and P143. The numbers below the lines indicate the number of recombinants between *qNPT1* and an adjacent marker. The candidate gene was predicted to generate two alternative transcripts. The arrowhead indicates the target site designed for CRISPR/Cas9-based genome editing. **(H)** Sequence variants at the *NPT1* locus in both the promoter and coding regions shown in **G**. The specific nucleotide variants of the *npt1* allele are indicated by the pink boxes.

To investigate genes that regulate the NPT architecture, we constructed a set of 670 recombinant inbred lines (RILs) derived from a cross between the *japonica* rice variety Chunjiang06 (CJ06) and a selected NPT line (IR66167-27-5-1-6). Among these RIL populations, one line (RIL52) exhibited the NPT phenotypes, consisting of reduced tiller number per plant, enhanced grain number per panicle and thickened culm (Figure 1A-1E). Using single nucleotide polymorphism (SNP) and insertion-deletion polymorphism (Indel) genotyping methods, a QTL analysis of a recurrent backcross (BC_2_F_2_) population, in which RIL52 was the donor parent and CJ06 was the recurrent parent, identified a major locus, *qNPT1* (*New Plant Type* *1*), as being pleiotropically responsible for the culm diameter, the tiller number per plant and the grain number per panicle (Figure 1F).

Positional cloning of *qNPT1* was performed using BC_2_F_2_ and BC_2_F_3_ progenies developed from a backcross between RIL52 (the donor parent) and the *indica* variety Zhefu802 (the recurrent parent). Co-segregation analysis of BC_2_F_2_ progeny suggested that a recessive *qnpt1* allele from IR66167-27-5-1-6 was responsible for the NPT architecture. The candidate region was narrowed to an ∼ 4.1 Kbp segment flanked by the markers P139 and P143, which harbors the promoter and part of the coding sequence of the gene at LOC_Os08g42540 (Figure 1G). The candidate gene is predicted to encode a deubiquitinating enzyme with homology to human OTUB1 (Supplementary information, Figure S1), a protein associated with the regulation of p53 stability and DNA damage repair^20,21,22,23^. On this basis, LOC_Os08g42540 is hereafter referred to as *OsOTUB1*. A sequence comparison revealed five nucleotide variations could distinguish the alleles responsible for the NPT phenotypes vs the conventional phenotypes (Figure 1H); three of these variations were SNPs in intronic regions, whereas one was an Indel, and one was a SNP in the promoter region. However, these nucleotide sequence variations do not change the amino acid sequence of the protein product.

### Downregulation of *OsOTUB1* is correlated with NPT architecture

**Figure 2:**
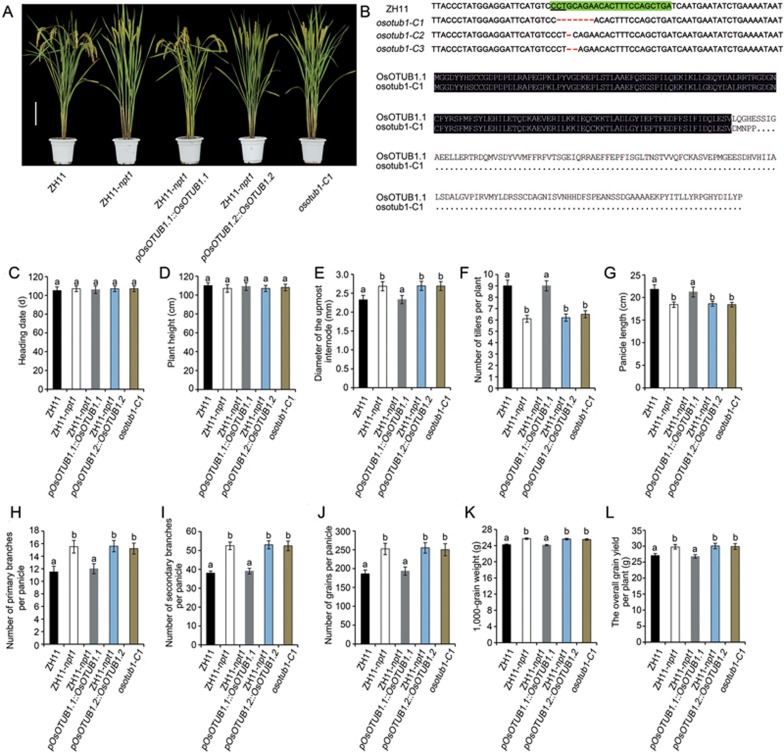
Effect of functional *OsOTUB1* on plant architecture and grain yield. **(A)** Mature plant morphology. Scale bar, 20 cm. **(B)** Loss-of-function mutations of *OsOTUB1* generated via CRISPR/Cas9-based genome editing. The target sequence indicated by the green boxes is located on the reverse strand of *OsOTUB1.1* shown in Figure 1G. The *osotub1-C1* mutant harbors a 7-bp deletion (in red) that results in a premature stop codon. **(C)** Heading date. **(D)** Plant height. **(E)** Diameter of the uppermost internode. **(F)** Number of tillers per plant. **(G)** Panicle length. **(H)** Number of primary branches per panicle. **(I)** Number of secondary branches per panicle. **(J)** Number of grains per panicle. **(K)** 1 000-grain weight. **(L)** Overall grain yield per plant. Data are shown as the mean ± SEM (*n* = 288). All phenotypic data were measured in paddy-grown plants under normal cultivation conditions. The presence of the same lowercase letter denotes a non-significant difference between means (*P* < 0.05).

**Figure 3:**
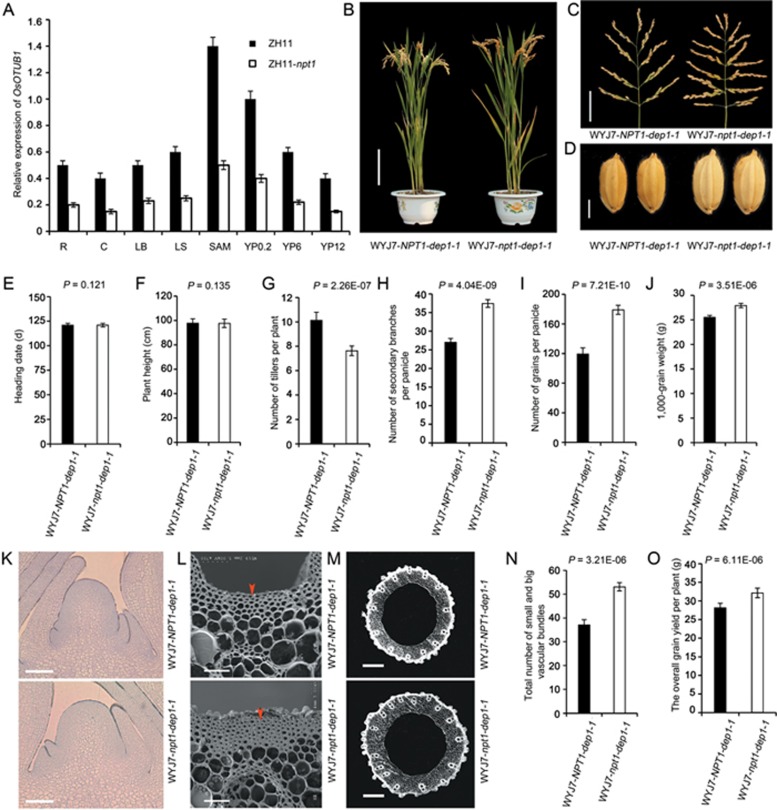
Pyramiding of the *npt1* and *dep1-1* alleles enhances panicle branching and grain yield in rice. **(A)** Levels of *OsOTUB1* transcript present in the organs of NIL plants. R, root; C, culm; LB, leaf blade; LS, leaf sheath; SAM, shoot apical meristem; YP0.2, YP6, YP12: young panicles, with a mean length of 0.2 cm, 6 cm or 12 cm, respectively. Relative expression levels are presented as the relative number of copies per 1 000 copies of rice *Actin1.* Data are shown as the mean ± SEM (*n* = 3). **(B)** Plant morphology. Scale bar, 20 cm. **(C)** Panicle morphology. Scale bar, 5 cm. **(D)** Grain morphology. Scale bar, 2 mm. **(E-J)** A quantitative comparison of the two NILs. **(E)** Heading date. **(F)** Plant height. **(G)** Number of tillers per plant. **(H)** Number of secondary branches per panicle. **(I)** Number of grains per panicle. **(J)** 1 000 grain weight. **(K)** An image of the shoot apical meristem. Scale bar, 50 μm. **(L)** Scanning electron microscopy image of a culm. Scale bar, 25 μm. **(M)** Culm vascular system. Scale bar, 500 μm. **(N)**, Total number of large and small vascular bundles shown in **M**. **(O)** Overall grain yield per plant. Data are shown as the mean ± SEM (*n* = 288). All phenotypic data were measured from paddy-grown plants under normal cultivation conditions. Student's *t*-test was used to generate the *P* values.

**Figure 4:**
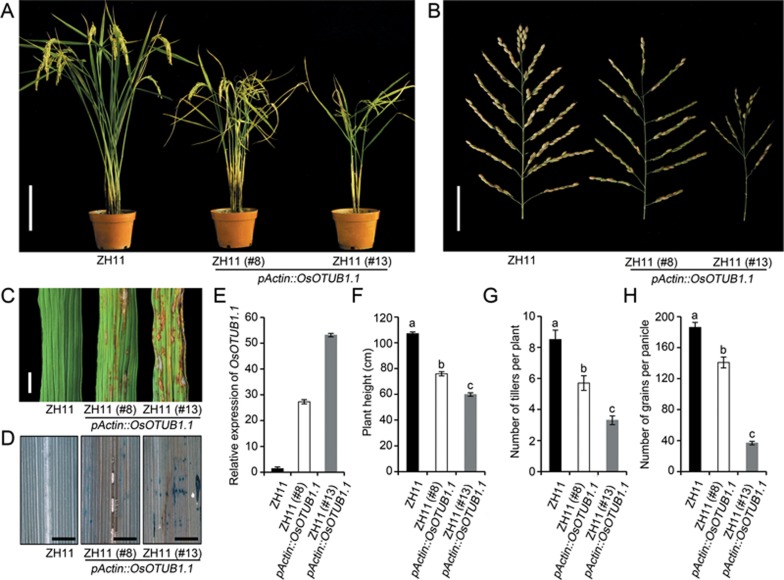
Phenotypes of transgenic ZH11 plants over-expressing *OsOTUB1.1*. **(A)** Two independent transgenic lines showed reduced tiller number and dwarfism. Scale bar, 10 cm. **(B)** Panicle size was reduced. Scale bar, 5 cm. **(C)** Leaves suffered from necrosis. Scale bar, 3 mm. **(D)** Apoptosis was induced in the flag leaves, assayed via Evans Blue staining. Scale bar, 3 mm. **(E)** Abundance of *OsOTUB1*.*1* transcript in the young panicle. Transcription relative to the level in ZH11 plants (set to one). Data are shown as the mean ± SEM (*n* = 3). **(F)** Plant height. **(G)** Number of tillers per plant. **(H)** Number of grains per panicle. Data are shown as the mean ± SEM (*n* = 60). All phenotypic data were measured in paddy-grown rice plants under normal cultivation conditions. The presence of the same lowercase letter denotes a non-significant difference between means (*P* < 0.05, panels **F** to **H**).

We next generated the near-isogenic line (NIL) ZH11-*npt1*, which harbors an ∼ 240 Kbp segment including the *npt1* allele from IR66167-27-5-1-6 in the background of the *japonica* rice variety Zhonghua11 (ZH11; Figure 2A). Quantitative RT-PCR analysis revealed that the peak abundance of the *OsOTUB1* transcript occurred in the shoot meristem and young panicles (Figure 3A). Histochemical promoter-GUS fusion assays showed that *OsOTUB1* was strongly expressed in vascular tissue as well as in the root cap and quiescent centre cells (Supplementary information, Figure S2). The abundance of the *OsOTUB1* transcript in ZH11-*npt1* was lower than that in ZH11 (Figure 3A). The phenotypes of *OsOTUB1* knockout mutants generated using CRISPR/Cas9^24^ (Figure 2A and 2B), were similar to those of ZH11-*npt1* plants, including reduced tiller number per plant, increased grain number per panicle, enhanced thousand-grain weight and a consequent increase in grain yield (Figure 2C-2L). In transgenic plants expressing *pOsOTUB1::OsOTUB1-GFP*, GFP signal was detectable in both the nucleus and the cytoplasm of root tip and leaf sheath cells (Supplementary information, Figure S3). Two alternatively spliced transcripts of *OsOTUB1* were predicted utilizing the Rice Annotation Project Database (Figure 1G). The phenotypes of transgenic ZH11-*npt1* plants expressing *OsOTUB1.1* cDNA driven by its native promoter were similar to those of ZH11 plants, whereas plants expressing *OsOTUB1.2* were similar to ZH11-*npt1* plants (Figure 2A and 2C-2L). These results suggest that *OsOTUB1.1* is functional, whereas *OsOTUB1.2* is non-functional. In addition, the transgenic ZH11 plants over-expressing *OsOTUB1.1* were dwarfed in stature (Figure 4A), set fewer grains and displayed leaf necrosis (Figure 4B-4H). Taken together, these results indicate that reduction- or loss-of-function alleles are associated with the formation of an ideotype architecture.

### Pyramiding of *npt1* and *dep1-1* alleles enhances grain yield in rice.

Haplotype analysis revealed that the *npt1* allele has not been exploited by rice breeders of elite *indica* and temperate *japonica* varieties (Figure 1H). Because the high-yielding *dep1-1* allele has been heavily used by Chinese breeders^25,26^, the effect of combining the *npt1* and *dep1-1* alleles was evaluated. NILs for allelic combinations of the *qNPT1* and *qDEP1* loci were generated in the elite *japonica* rice variety Wuyunjing7 that carries the *NPT1 and dep1-1* alleles and is hereafter referred to as WYJ7-*NPT1-dep1-1*. The WYJ7-*npt1-dep1-1* and WYJ7-*NPT1-dep1-1* plants did not differ from one another with respect to the heading date and plant height (Figure 3B, 3E and 3F), whereas WYJ7-*npt1-dep1-1* plants formed fewer tillers and set a larger number of heavier grains than WYJ7-*NPT1-dep1-1* plants (Figure 3G-3J). The shoot apical meristems formed in WYJ7-*npt1-dep1-1* plants were larger than those formed in WYJ7-*NPT1-dep1-1* plants (Figure 3K), which implied that *npt1* and *dep1-1* acted synergistically to enhance meristematic activity^5^. The WYJ7-*npt1-dep1-1* plants were also characterized by a greater number of vascular bundles, thicker culm, parenchyma and sclerenchyma cell walls (Figure 3L-3N). Importantly, over three successive seasons, the overall grain yield of the WYJ7-*npt1-dep1-1* plants was 10.4% higher than that of the WYJ7-*NPT1-dep1-1* plants (Figure 3O). Therefore, pyramiding of the *npt1* and *dep1-1* alleles represents an effective means of boosting grain yield in rice.

### OsOTUB1 has canonical deubiquitinase activity

**Figure 5:**
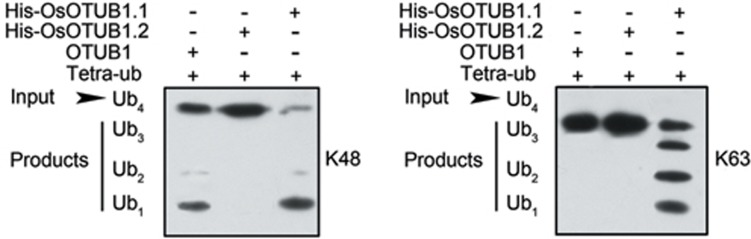
OsOTUB1 displayed cleavage activity for K48- and K63-linked ubiquitin tetramers. Cleavage activity towards K48- and K63-linked ubiquitin tetramers (Tetra-ub) was analysed using OTUB1, His-OsOTUB1.1 or OsOTUB1.2. The inputs (Ub_4_) and their cleavage products (trimers (Ub_3_), dimers (Ub_2_) and monomers (Ub_1_)) are labelled on the left. The products were visualised via western blotting using an anti-ubiquitin antibody.

Given the predicted deubiquitinase activity of OsOTUB1, we next examined its ability to cleave linear K48- and K63-linked tetra-ubiquitin. Consistent with the behaviour of its human homologue, OsOTUB1 showed strong cleavage activity when presented with K48-linked tetra-ubiquitin^27,28^, but unlike OTUB1, it also displayed a moderate level of activity when presented with K63-linked forms (Figure 5). ZH11-*npt1* plants expressing either *OTUB1* or its orthologue from mouse, maize or barley under the control of the rice *Actin* promoter exhibited phenotypes indistinguishable from those of ZH11 plants or transgenic ZH11-*npt1* plants expressing *OsOTUB1.1* (Supplementary information, Figure S4). These results suggest that *OsOTUB1* and its orthologues from the four examined species are functionally interchangeable.

**Figure 6:**
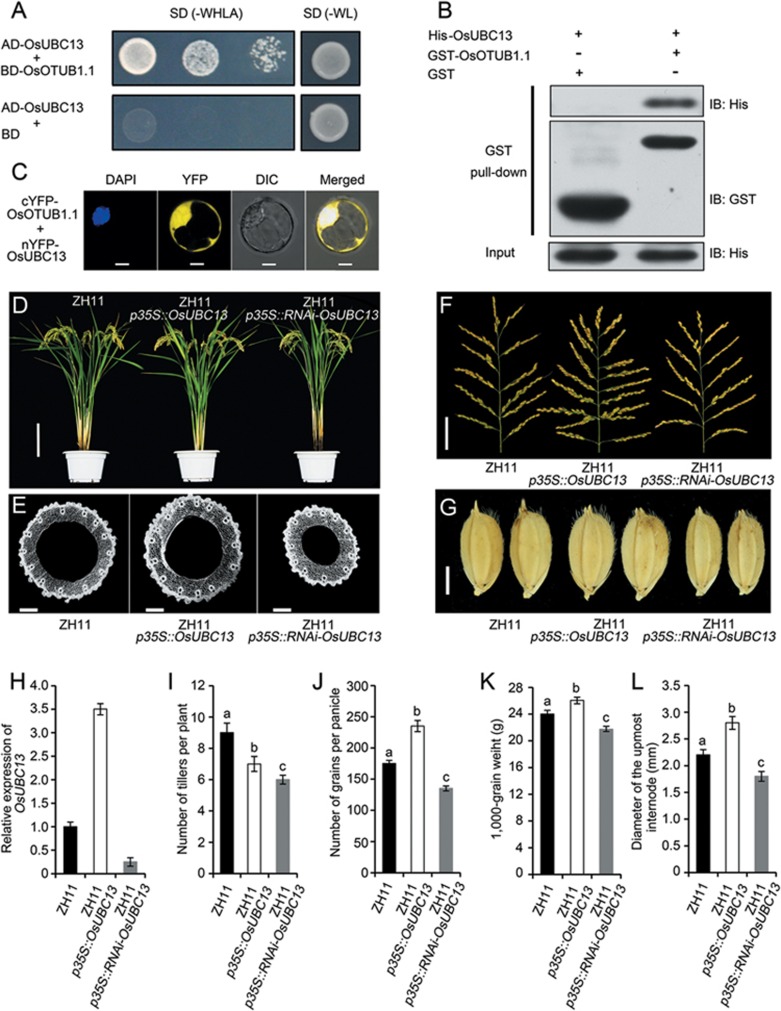
The interaction between OsOTUB1 and OsUBC13 proteins regulates plant architecture. **(A)** Yeast two-hybrid assays. **(B)** Pull-down assays using recombinant GST-OsOTUB1 and His-OsUBC13. **(C)** BiFC assays in rice protoplasts. Scale bar, 10 μm. **(D)** Morphology of transgenic ZH11 plants. Scale bar, 20 cm. **(E)** Cross-section of the uppermost internodes. Scale bar, 500 μm. **(F)** Effect of *OsUBC13* on panicle branching. Scale bar, 5 cm. **(G)** Grain size and shape. Scale bar, 2 mm. **(H)** Abundance of the *OsOTUB1* transcript in young panicles relative to the level in ZH11. Data are shown as the mean ± SEM (*n* = 3). **(I)** Number of tillers per plant. **(J)** Number of grains per panicle. **(K)** 1 000-grain weight. **(L)** Diameter of the uppermost internode. Data are shown as the mean ± SEM (*n* = 30). All phenotypic data were measured in paddy-grown rice plants under normal cultivation conditions. The presence of the same lowercase letter denotes a non-significant difference between means (*P* < 0.05, panels **I** to **L**).

OTUB1 is a deubiquitinating enzyme that forms a complex *in vivo* with E2 ubiquitin-conjugating enzymes. The OTUB1 protein interacts directly with the E2 ubiquitin-conjugating protein UBC13 and prevents ubiquitin transfer, thereby inhibiting double-strand-break-induced chromatin ubiquitination^20,21^. Similarly, interactions were detected *in vitro* and in *vivo* between the rice OsOTUB1 and OsUBC13 proteins (Figure 6A-6C). Overexpression of *OsUBC13* resulted in phenotypes similar to those of plants carrying the *npt1* allele, at least with respect to the number of grains per panicle, the number of tillers per plant and the culm diameter (Figure 6D-6L). In contrast, silencing of *OsUBC13* by RNAi resulted in phenotypes reminiscent of plants in which *OsOTUB1.1* was over-expressed (Figure 4F-4H and Figure 6D-6L).

### OsOTUB1 interacts with SPL transcription factors

**Figure 7:**
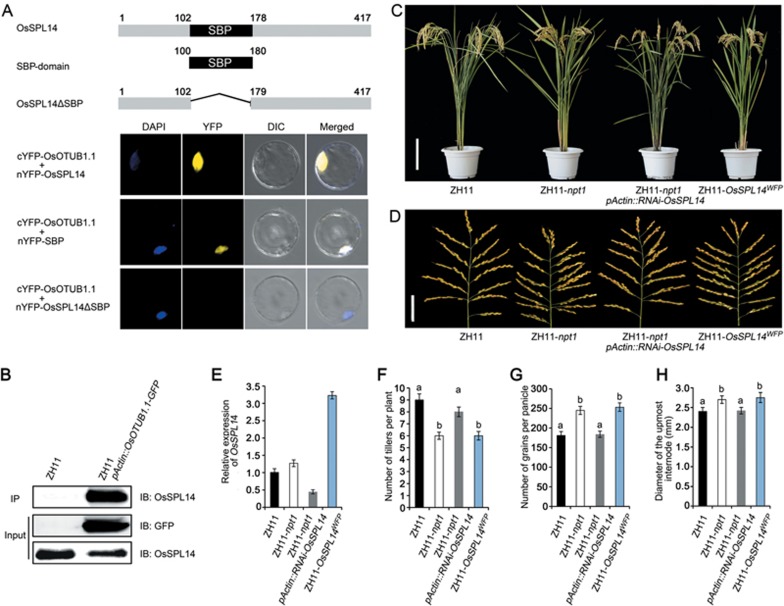
The OsOTUB1-OsSPL14 interaction controls plant architecture. **(A)** BiFC assays. The N-terminus of YFP-tagged OsSPL14, the SBP domain or a deleted version of OsSPL14 was co-transformed into rice protoplasts along with the C-terminus of YFP-tagged OsOTUB1.1. Panels (from left to right), DAPI staining, YFP signal, differential interference contrast image, merged channels. Scale bar, 10 μm. **(B)** Co-immunoprecipitation of OsOTUB1.1-GFP and OsSPL14. IB, Immunoblot; IP, immunoprecipitation. **(C)** Plant morphology. Scale bar, 20 cm. **(D)** Panicle morphology. Scale bar, 5 cm. **(E)** *OsSPL14* transcript abundance. Transcription relative to the level in ZH11 plants was set to one. Data are shown as the mean ± SEM (*n* = 3). **(F)** Number of tillers per plant. **(G)** Number of grains per panicle. **(H)** Culm diameter. All phenotypic data were measured in field-grown plants under normal cultivation conditions. Data in **F**-**H** are shown as the mean ± SEM (*n* = 120). The presence of the same lowercase letter denotes a non-significant difference between means (*P* < 0.05).

A yeast two-hybrid screen targeting proteins that interact with OsOTUB1 identified 72 candidate interactors (Supplementary information, Table S1), including a rice homologue of the SQUAMOSA promoter-binding protein-like (SPL) transcription factor OsSPL14, which is known to control plant architecture associated with reduced tiller number, thickened culm and enhanced grain number^17,18^. Bimolecular fluorescence complementation (BiFC) and co-immunoprecipitation assays showed that the OsSPL14-OsOTUB1 interaction clearly occurred *in planta* (Figure 7A and 7B). A deletion analysis revealed that the conserved squamosa promoter-binding protein (SBP) domain was both necessary and sufficient for the *in vitro* and *in vivo* interactions (Figure 7A and Supplementary information, Figure S5). Furthermore, BiFC assays demonstrated that OsOTUB1 was able to interact with the full set of rice SPL transcription factors (Supplementary information, Figure S6), consistent with data showing that the abundance of *OsSPL7*^29^, *OsSPL13*^30^, *OsSPL14*^17,18^ or *OsSPL16*^13,14^ transcript is correlated with one or multiple of the following phenotypes: reduced tiller number, increased grain number and enhanced grain weight^31^.

**Figure 8:**
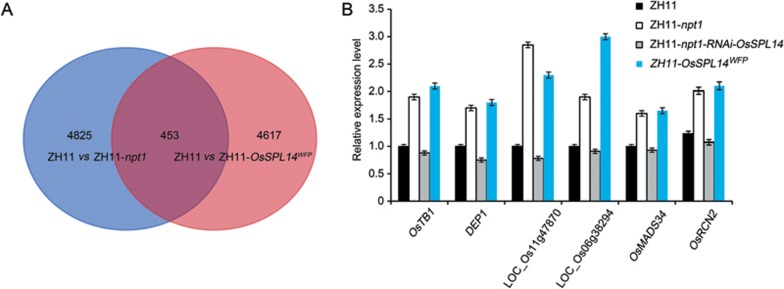
*OsOTUB1* and *OsSPL14* antagonistically regulate common target genes. **(A)** Number and overlap of *OsSPL14*-activated and *OsOTUB1*-repressed target genes. RNA-seq was performed using young panicles (< 0.2 cm in length) of the NIL plants. **(B)** The abundances of *OsSPL14*-regulated genes examined in young panicles relative to the levels in ZH11. Data are shown as the mean ± SEM (*n* = 3).

Previous studies have shown that rice plants carrying the *OsSPL14^WFP^* allele display increased expression of *OsSPL14*^18^. We found that the presence of either the *npt1* or *OsSPL14^WFP^* allele was associated with the formation of the NPT architecture, whereas the phenotypes of ZH11-*npt1* plants in which *OsSPL14* had been silenced by RNAi were similar to those of ZH11 plants (Figure 7C-7H). This result suggests that the NPT architecture of the *npt1* allele is dependent on the function of *OsSPL14*. Comparative RNA-seq-based transcriptomic analysis of ZH11, ZH11-*npt1* and ZH11-*OsSPL14^WFP^* revealed that the transcript levels of 453 common target genes were higher in both ZH11-*npt1* and ZH11-*OsSPL14^WFP^* than in ZH11 (Figure 8A and Supplementary information, Table S2), which was validated using quantitative real-time PCR (qRT-PCR)^14,30,32^. In contrast, the abundances of the examined target genes were greatly reduced in ZH11-*npt1* plants in which *OsSPL14* was silenced (Figure 8B). EMSAs revealed that the OsOTUB1-OsSPL14 interaction was unlikely to affect the binding affinity of OsSPL14 for its target GTAC motifs (Supplementary information, Figure S7). Taken together, these results reveal that *OsOTUB1* and *OsSPL14* act antagonistically to control plant architecture by regulating a common set of target genes.

### Effect of OsOTUB1-dependent K63-linked ubiquitination on OsSPL14 protein stability

**Figure 9:**
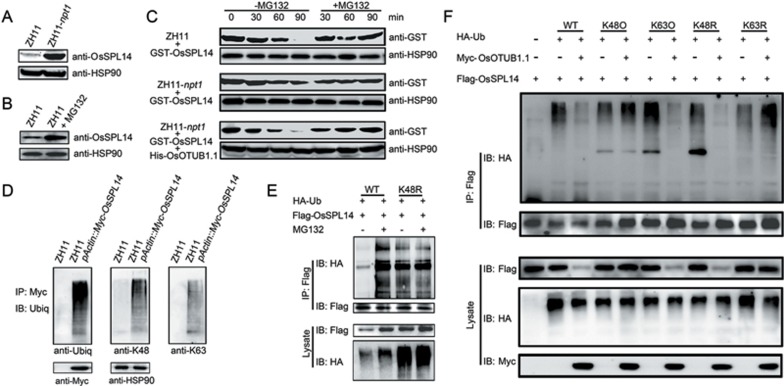
OsOTUB1 promotes the degradation of OsSPL14. **(A)** Accumulation of OsSPL14 in ZH11 and ZH11-*npt1* plants. The abundance of the HSP90 protein was used as a loading control. **(B)** Treatment with the proteasome inhibitor MG132 stabilizes OsSPL14. Total protein was extracted from young panicles (< 0.2 cm in length) of ZH11 plants exposed to either 0 or 50 μM MG132. The immunoblot was probed with either anti-OsSPL14 or anti-HSP90 antibodies. **(C)** OsOTUB1 destabilizes OsSPL14. The lysates from young panicles of ZH11 and ZH11-*npt1* plants were co-incubated with GST-OsSPL14 in the presence or absence of His-OsOTUB1. The lysates were harvested at various times and immunoblotted to assess the accumulation of OsSPL14 and HSP90. **(D)** Ubiquitination of OsSPL14. The protein extracts from young panicles were immunoprecipitated using an anti-Myc antibody, then analysed using anti-ubiquitin, anti-K48-linked ubiquitin or anti-K63-linked ubiquitin chain conjugates. **(E)** Flag-OsSPL14 can be modified via K48-ubiquitin linkage. Rice protoplasts were co-transfected with Flag-OsSPL14 and HA-ubiquitin (either HA-tagged WT or K48R ubiquitins), and the ubiquitinated forms of Flag-OsSPL14 were immunoprecipitated using an anti-Flag antibody and then analysed using an anti-HA antibody. **(F)** The K63-linked ubiquitination of OsSPL14 is regulated by OsOTUB1. Rice protoplasts were co-transfected with Flag-OsSPL14 and HA-ubiquitin (either HA-tagged WT, K48R, K63R, K48O or K63O ubiquitins) in the presence or absence of Myc-OsOTUB1; lysates were then harvested and immunoblotted to assess the accumulation of OsSPL14 and analysed for ubiquitinated forms of Flag-OsSPL14, as described in **E**.

There was no difference in the abundance of the *OsSPL14* transcript between ZH11 and ZH11-*npt1* (Figure 7E), but the accumulation of OsSPL14 was much higher in the latter genotype (Figure 9A). When exposed to the proteasome inhibitor MG132, OsSPL14 accumulation was obviously increased in ZH11 (Figure 9B). These results suggest that OsOTUB1 (unlike OTUB1, which regulates the stability of p53 and SMAD proteins^22,23^) promotes the degradation of OsSPL14. When lysates prepared from young panicles of ZH11 were challenged with GST-OsSPL14, the amount of GST-OsSPL14 decreased over time, but GST-OsSPL14 degradation was inhibited when an MG132 treatment was included (Figure 9C). The stable accumulation of GST-OsSPL14 was higher when the lysates were prepared from ZH11-*npt1* than from ZH11 panicles, whereas the degradation of GST-OsSPL14 was accelerated in lysates from ZH11-*npt1* panicles in the presence of His-OsOTUB1 (Figure 9C). Western blotting analysis showed that it was possible to detect polyubiquitinated forms of Myc-OsSPL14 immunoprecipitated from young panicles using antibodies that recognize total ubiquitin, K48-linked polyubiquitin or K63-linked polyubiquitin (Figure 9D). The implication of these results is that OsSPL14 is modulated by both K48- and K63-linked ubiquitination^33^.

The analysis was extended to investigate endogenous E3 ligase-mediated ubiquitination of OsSPL14. In the presence of WT ubiquitin, treating rice protoplasts expressing Flag-OsSPL14 with MG132 resulted in enhanced accumulation of polyubiquitinated Flag-OsSPL14; however, in the presence of K48R-ubiquitins, there was no perceptible effect of MG132 treatment on the accumulation of polyubiquitinated Flag-OsSPL14 (Figure 9E). This observation is consistent with the notion that K48-linked ubiquitination (K48Ub) of OsSPL14 is required for its proteasome-mediated degradation. In the presence of Myc-OsOTUB1.1, the amount of ubiquitinated Flag-OsSPL14 was clearly lower in the presence of either K48R or K63O mutant ubiquitins, but it remained unaffected in the presence of either K48O or K63R mutant ubiquitins (Figure 9F). Moreover, Myc-OsOTUB1.1 promoted the degradation of Flag-OsSPL14 only in the presence of WT, K63O or K48R ubiquitins (Figure 9F), indicating that the stabilization of OsSPL14 is correlated with K63-linked ubiquitination. Collectively, these results suggest that OsOTUB1-mediated inhibition of the K63-linked ubiquitination of OsSPL14 is required for its proteasome-dependent degradation.

## Discussion

Because of the rapid increase in world population and the gradual decrease in arable land, food shortage is becoming a serious global problem. Improvement of grain productivity has been the key focus of rice breeding programmes in many countries over the past 50 years. There have been two quantum leaps regarding the grain yield potential of rice^4^. The first was brought about by the development of semi-dwarf varieties in the late 1950s in China and in the early 1960s at the IRRI^1,2^. The second leap in the improvement of yield potential was conferred by the exploitation of heterosis in the 1970s in China^3^. To overcome the yield ceiling of current rice varieties, an 'ideotype approach' to improve yield potential via selection for optimal plant architecture has been proposed in rice breeding programmes in China and at the IRRI^4^.

Since the 1980s, China's 'super' rice breeding project has developed a number of high-yielding *japonica* rice varieties whose architecture is characterised by dense and erect panicles, which is controlled by the *DEP1* locus^5^. Rice plants carrying a gain-of-function *dep1-1* allele display dense and erect panicle architecture, increased number of grains per panicle and increased grain yield^25^. In the late 1980s and early 1990s, IRRI scientists proposed NPT rice, whose architecture is characterized by larger panicles, fewer sterile tillers and stronger culms than conventional varieties^4^. Although several NPT rice strains have been released as commercial varieties, the genetic basis of the NPT traits has only been explained as being associated with a QTL, without any understanding of the underlying molecular mechanism^6,7,8^.

In this study, we showed that a major QTL, *qNPT1*, is responsible for the NPT architecture, and it encodes a deubiquitinating enzyme with homology to human OTUB1. Our results suggest that downregulation of *OsOTUB1* enhances meristematic activity (Figure 3K), resulting in the formation of an improved plant architecture with reduced tiller number per plant, increased grain number per panicle, enhanced grain weight and a consequent increase in grain yield (Figure 2). Similar results were observed in *OsOTUB1* loss-of-function plants (Figure 2). In contrast, constitutive expression of *OsOTUB1* resulted in fewer grains per panicle, indicating that *OsOTUB1* plays a negative role in the regulation of panicle branching and grain yield in rice. In addition, we showed that the rice *OsOTUB1* gene and its orthologues from other cereal crops are functionally interchangeable. Therefore, manipulation of the transcript level of *OsOTUB1* orthologues in other cereal crops through genome engineering using the CRISPR/Cas9 system may also be applicable to facilitation of the breeding of new varieties that can increase crop production.

A haplotype analysis of the *NPT1* locus revealed that the *npt1* allele from the NPT rice strains has not been used to breed elite *indica* and temperate *japonica* varieties. Because the high-yielding *dep1-1* allele has been widely used in *japonica* rice breeding programmes in China^5,25^, we employed QTL pyramiding based on combinations of the *npt1* and *dep1-1* alleles with molecular marker-assisted selection. Introduction of the *npt1* allele into the high-yielding rice variety WYJ7 carrying the *dep1-1* allele resulted in increased grain productivity, without changes in plant height (Figure 3). These results indicate that the *npt1* allele is correlated with the formation of the NPT architecture, resulting in larger panicles, fewer sterile tillers and stronger culms. More importantly, we demonstrated that pyramiding of the high-yielding *npt1* and *dep1-1* alleles represents a new strategy for increasing rice yield potential above what is currently achievable.

The human OTUB1 protein functions as an atypical deubiquitinating enzyme^20,21^, with strict specificity for K48-linked ubiquitin chains, and stabilizes its target proteins, such as p53 and SMAD2/3^22,23^. Unlike OTUB1, the rice OsOTUB1 protein exhibits detectable cleavage activity for both K48-linked and K63-linked ubiquitin chains. Furthermore, OsOTUB1 genetically and physically interacts with the E2 ubiquitin-conjugating protein OsUBC13, and OsOTUB1 exhibits canonical deubiquitinase activity. Previous studies have shown that higher expression of *OsSPL14* inhibits shoot branching but promotes panicle branching, resulting in an ideal plant architecture with respect to reduced tiller number, increased grain number and thickened culm^17,18^. In addition, the RING-finger E3 ligase IPI1 modulates the stability of OsSPL14 by adding K48-linked polyubiquitin chains during the reproductive stage and K63-linked polyubiquitin chains during the vegetative stage^33^. We report here that OsOTUB1 interacts directly with the full set of rice SPL transcription factors. Our results suggest that K48-linked ubiquitination of OsSPL14 is required for its proteasome-mediated degradation, but OsOTUB1 could limit the K63-linked ubiquitination of OsSPL14 and promote the K48Ub-dependent proteasomal degradation of OsSPL14. In mammalian cells, ESCRT0 protein complexes have been shown to selectively associate with K63-linked chains and block their binding to 26S proteasomes^34^. Further studies will be essential to define the fate of nuclear K63-ubiquitinated OsSPL14 at various developmental stages and to reveal whether similar mechanisms involved in specific ubiquitin-binding domain proteins protect these conjugates from proteasomal degradation, as occurs in the cytosol of mammalian cells.

In summary, our results reveal that OsOTUB1 promotes the proteasomal degradation of OsSPL14, and reduced expression or loss-of-function of *OsOTUB1* results in increased accumulation of OsSPL14, which in turn defines the NPT architecture associated with increased grain yield. Indeed, the miR156-targeted SBP domain transcription factors play important roles in the regulation of stem cell function and flowering in plants^35,36,37^. Non-canonical OsOTUB1-mediated regulation of SBP domain transcription factors establishes a new framework for studying meristem cell fate, inflorescence architecture and flower development. Our findings shed light on the molecular basis of an ideotype approach in breeding programmes. Manipulation of the *miR156-OsSPL14-OsOTUB1* regulatory module also provides a potential strategy for facilitating the breeding of new rice varieties with higher grain productivity.

## Materials and Methods

### Plant materials and growing conditions

A population of 670 RILs was bred from a cross between the Chinese temperate *japonica* rice variety Chunjiang06 and the NPT selection IR66167-27-5-1–6. The rice accessions used for sequence diversity analysis have been described elsewhere^13,25^. NIL plants carrying *npt1*, *OsSPL14^WFP^*^18,38^, or allelic combinations of the *qNPT1* and *qDEP1* loci were bred by crossing RIL52 seven times with either Zhonghua11 or Wuyunjing7. Paddy-grown plants were spaced 20 cm apart and were grown during the standard growing season at three experimental stations: one in Lingshui (Hainan Province), one in Hefei (Anhui Province) and one in Beijing. The primer sequences used for positional cloning and genotyping analysis are provided in Supplementary information, Table S3.

### Transgene constructs

The *OsOTUB1.1* and *OsOTUB1.2* coding sequences and their UTRs (5′: from the transcription start site to −2.5 Kbp; 3′: 1.5 Kbp downstream of the termination site) were amplified from ZH11 genomic DNA and introduced into the pCAMBIA2300 vector (CAMBIA) to generate *pOsOTUB1.1::OsOTUB1.1* and *pOsOTUB1.2::OsOTUB1.2*. The full-length cDNAs of *OsOTUB1* and its orthologues from other species (e.g., human, mouse, barley and maize) were amplified from the relevant cDNA template and then subcloned into the pActin::nos vector^25^, while *OsOTUB1.1* cDNA and its 5′UTR was introduced into the p35S::GFP-nos vector^22^ to generate the *pOsOTUB1.1::OsOTUB1.1-GFP-nos* construct. To generate *pCaMV35S::OsUBC13*, the full-length cDNA of *OsUBC13* was amplified from a ZH11 cDNA template and cloned into the pCaMV35S::nos vector. To generate *pCaMV35S::Myc-OsSPL14*, *OsSPL14* cDNA was amplified from a ZH11 cDNA template and cloned into pCaMV35S::Myc-nos. The gRNA constructs for the CRISPR/Cas9-mediated knockout of *OsOTUB1* were generated as described elsewhere^24^. A 300 bp fragment of *OsSPL14* cDNA and a 300 bp fragment of *OsUBC13* cDNA were amplified from ZH11 cDNA and used to construct the *pActin::RNAi-OsSPL14* and *pCaMV35S::RNAi-OsUBC13* transgenes as described elsewhere^25^. The transgenic rice plants were generated via *Agrobacterium*-mediated transformation as previously described^5^. The relevant primer sequences are presented in Supplementary information, Table S4.

### Quantitative real-time PCR analysis

Total RNA was extracted from plant tissues using the TRIzol reagent (Invitrogen), and treated with RNase-free DNase I (Invitrogen) according to the manufacturer's protocol. The resulting RNA was reverse-transcribed using a cDNA synthesis kit (TRANSGEN). Subsequent qRT-PCR was performed as described elsewhere^13^, including three independent RNA preparations as biological replicates. The rice *Actin1* gene was employed as a reference. The relevant primer sequences are presented in Supplementary information, Table S4.

### Yeast two-hybrid assays

Yeast two-hybrid assays were performed as described elsewhere^14,22^. The full-length *OsOTUB1.1* cDNA and an *OsOTUB1* C-terminal fragment were amplified from ZH11 cDNA and inserted into pGBKT7 (Takara Bio Inc.), and the full-length *OsUBC13* cDNA and a C-terminal fragment of *OsSPL14* were inserted into pGADT7 (Takara Bio Inc.). Each of these plasmids was validated by sequencing before being transformed into yeast strain AH109. The β-galactosidase assays were performed according to the manufacturer's protocol (Takara Bio Inc.). Cells harboring either an empty pGBKT7 or an empty pGADT7 were used as negative controls. The entire *OsOTUB1* sequence or a C-terminal fragment were used as the bait to screen a cDNA library prepared from poly(A)-containing RNA isolated from rice young panicles (< 0.2 cm in length). The experimental procedures for screening and plasmid isolation followed the manufacturer's protocol (Takara Bio Inc.). The relevant primer sequences are listed in Supplementary information, Table S4.

### BiFC assays

The full-length cDNAs of *OsOTUB1.1*, *OsUBC13*, *OsSPL1* through *OsSPL13* and *OsSPL15* through *OsSPL19*, along with both deleted and non-deleted versions of *OsSPL14*, were amplified from ZH11 cDNA, and the amplicons were inserted into the pSY-735-35S-cYFP-HA or pSY-736-35S-nYFP-EE vector^39^ to generate a set of fusion constructs. Two vectors for testing protein-protein interactions (e.g., nYFP-OsSPL14 and cYFP-OsOTUB1) were then co-transfected into rice protoplasts. After incubation in the dark for 14 h, the YFP signal was examined and photographed under a confocal microscope (Zeiss LSM710) as described elsewhere^25^. Each BiFC assay was repeated at least three times. The relevant primer sequences are listed in Supplementary information, Table S4.

### *In vitro* pull-down

The recombinant GST-OsOTUB1 fusion protein was immobilized on glutathione Sepharose beads and incubated with His-OsUBC13 for 30 min at 4 °C. The glutathione Sepharose beads were subsequently washed three times, followed by elution with elution buffer (50 mM Tris-HCl, 10 mM reduced glutathione, pH 8.0). The supernatant was subjected to immunoblotting analysis using anti-His and anti-GST antibodies (Santa Cruz).

### Co-immunoprecipitation and western blotting

Myc-OsSPL14 was extracted from young panicles (< 0.2 cm in length) of transgenic ZH11 plants harboring *pActin::Myc-OsSPL14* using a buffer composed of 50 mM HEPES (pH 7.5), 150 mM KCl, 1 mM EDTA, 0.5% Triton-X 100, 1 mM DTT and a proteinase inhibitor cocktail (Roche LifeScience, Basel, Switzerland). Agarose-conjugated anti-Myc antibodies (Sigma-Aldrich) were then added, and the reaction was held at 4 °C for at least 4 h, followed by washing 5∼6 times with TBS-T buffer and elution with 2× loading buffer. The obtained immunoprecipitates and lysates were subjected to SDS-PAGE, and the separated proteins were transferred to a nitrocellulose membrane (GE Healthcare). The Myc-OsSPL14 fusion proteins were then detected by probing the membrane with an anti-Myc antibody (Santa Cruz), and polyubiquitinated forms were detected by probing with antibodies that recognize total ubiquitin conjugates, antibodies that specifically recognize K48-polyubiquitin conjugates, or antibodies that specifically recognize K63-polyubiquitin conjugates (Abcam).

### Analysis of OsSPL14 degradation

Lysates obtained from young panicles (< 0.2 cm in length) harvested from ZH11 and ZH11-*npt1* plants were incubated with appropriate recombinant GST-OsSPL14 fusion protein in the presence or absence of the recombinant His-OsOTUB1 fusion protein. Protein was extracted from lysates that had either been exposed or not to 50 μM MG132 for a series of incubation times and then subjected to SDS-PAGE and western blotting using an anti-GST antibody (Santa Cruz). As a loading control, the abundance of HSP90 was detected by probing with an anti-HSP90 antibody (BGI). The lysis buffer contained 25 mM Tris-HCl (pH 7.5), 10 mM NaCl, 10 mM MgCl_2_, 4 mM PMSF, 5 mM DTT and 10 mM ATP as described elsewhere^40^.

### Linear K48- and K63-linked tetra-ubiquitin cleavage assays

An ∼1 μg aliquot of recombinant GST-OsOTUB1.1, GST-OsOTUB1.2 or OTUB1 was added to 20 μL of 50 mM Tris-HCl (pH 7.4), 150 mM NaCl, 0.5 mM dithiothreitol containing 2.5 μg of linear K48- and K63-linked tetra-ubiquitin (Boston Biochem) and held for 1 h at 37 °C. The reaction products were analysed via western blotting using an anti-ubiquitin antibody (Abcam) as described elsewhere^27^.

### Analysis of *in vitro* ubiquitination

Rice protoplasts prepared from young panicles of ZH11-*npt1* plants (< 0.2 cm in length) were transfected with the plasmids *pUC19-35S-Flag-OsSPL14-RBS*^41^ and *pUC19-35S-HA-Ubiq-RBS* (HA-tagged ubiquitin (WT), K48R (K48 mutated to arginine), K63R (K63 mutated to arginine), K48O (ubiquitin with only K48, with the other lysine residues mutated to arginine), or K63O (ubiquitin with only K63, with the other lysine residues mutated to arginine)), in the presence or absence of the *pUC19-35S-Myc-OsOTUB1-RBS* plasmid. After 15 h, the protoplasts were lysed in extraction buffer (50 mM Tris-HCl (pH 7.4), 150 mM KCl, 1 mM EDTA, 0.5% Triton-X 100, 1 mM DTT) containing a proteinase inhibitor cocktail (Roche LifeScience). The resulting lysates were incubated with agarose-conjugated anti-Flag antibodies (Sigma-Aldrich) for at least 4 h at 4 °C, then rinsed 5-6 times in extraction buffer and eluted with 3× Flag peptide (Sigma-Aldrich). The obtained immunoprecipitates were separated via SDS-PAGE and transferred to a nitrocellulose membrane (GE Healthcare), which was used for western blotting analysis with anti-HA and anti-Flag conjugate antibodies (Sigma-Aldrich).

### Statistical analysis

Statistical analysis was performed by one-way ANOVA or unpaired Student's *t*-test. *P*-values of < 0.05 were considered to indicate statistical significance. Statistical calculations were performed using Microsoft Excel 2010.

## Author Contributions

SW performed most of the experiments; SW, KW and QQ conducted the QTL analysis and positional cloning; KW, YW and SW constructed the NILs; SW, QL and YP performed field experiments; SW, QL, XL and JW performed the yeast two-hybrid screen; SW, KW, JZ and SL characterised the phenotypes of transgenic plants; SW, KW and YY analysed grain yield. XF designed the experiments and wrote the manuscript. All authors have discussed the results and contributed to the drafting of the manuscript.

## Competing Financial Interests

The authors declare no competing financial interests.

## Supplementary Information

Supplementary information, Figure S1 Protein sequence alignment.

Supplementary information, Figure S2 Analysis of *pOsOTUB1::GUS* expression.

Supplementary information, Figure S3 Subcellular localization of OsOTUB1.1-GFP.

Supplementary information, Figure S4 Effects of expressing human *OTUB1* or its orthologues on ZH11-*npt1* plant architecture.

Supplementary information, Figure S5 The SBP domain is required for the OsSPL14-OsOTUB1 interaction.

Supplementary information, Figure S6 OsOTUB1 interacts with rice SPL transcription factors.

Supplementary information, Figure S7 Effect of the OsSPL14-OsOTUB1 interaction on the DNA binding affinity of OsSPL14.

Supplementary information, Table S1 Identification of the putative OsOTUB1-interacting proteins using yeast two-hybrid assays.

Supplementary information, Table S2 The common target genes regulated by *npt1* and *OsSPL14^WFP^* alleles.

Supplementary information, Table S3 The primer sequences used for map-based cloning and genotyping assays.

Supplementary information, Table S4 The primer sequences used for DNA constructs and transcript analysis.

## References

[bib1] Sasaki A, Ashikari M, Ueguchi-Tanaka M, et al. Green revolution: a mutant gibberellin-synthesis gene in rice. Nature 2002; 416:701–702. 11961544 10.1038/416701a

[bib2] Spielmeyer W, Ellis MH, Chandler PM. Semidwarf (sd-1) “green revolution” rice, contains a defective gibberellin 20-oxidase gene. Proc Natl Acad Sci USA 2002; 99:9043–9048. 12077303 10.1073/pnas.132266399 PMC124420

[bib3] Yuan L. Hybrid rice breeding for super high yield. Hybrid Rice 1997; 12:1–6.

[bib4] Khush GS. Breaking the yield frontier of rice. Geo J 1995; 35:329–332.

[bib5] Huang X, Qian Q, Liu Z, et al. Natural variation at the DEP1 locus enhances grain yield in rice. Nat Genet 2009; 41:494–497. 19305410 10.1038/ng.352

[bib6] Khush GS. Strategies for increasing the yield potential of cereals: case of rice as an example. Plant Breeding 2013; 132:433–436.

[bib7] Marathi B, Guleria S, Mohapatra T, et al. QTL analysis of novel genomic regions associated with yield and yield related traits in new plant type based recombinant inbred lines of rice (*Oryza sativa* L.). BMC Plant Biol 2012; 12:137. 22876968 10.1186/1471-2229-12-137 PMC3438134

[bib8] Peng S, Khush GS, Virk P, Tang Q, Zou Y. Progress in ideotype breeding to increase rice yield potential. Field Crops Res 2008; 108:32–38.

[bib9] Ashikari M, Sakakibara H, Lin S, et al. Cytokinin oxidase regulates rice grain production. Science 2005; 309:741–745. 15976269 10.1126/science.1113373

[bib10] Wu Y, Wang Y, Mi X, et al. The QTL GNP1 encodes GA20ox1, which increases grain number and yield by increasing cytokinin activity in rice panicle meristems. PLoS Genet 2016; 12:e1006386. 27764111 10.1371/journal.pgen.1006386 PMC5072697

[bib11] Fan C, Xing Y, Mao H, et al. GS3, a major QTL for grain length and weight and minor QTL for grain width and thickness in rice, encodes a putative transmembrane protein. Theor Appl Genet 2006; 112:1164–1171. 16453132 10.1007/s00122-006-0218-1

[bib12] Ishimaru K, Hirotsu N, Madoka Y, et al. Loss of function of the IAA-glucose hydrolase gene TGW6 enhances rice grain weight and increases yield. Nat Genet 2013; 45:707–711. 23583977 10.1038/ng.2612

[bib13] Wang S, Wu K, Yuan Q, et al. Control of grain size, shape and quality by OsSPL16 in rice. Nat Genet 2012; 44:950–954. 22729225 10.1038/ng.2327

[bib14] Wang S, Li S, Liu Q, et al. The OsSPL16-GW7 regulatory module determines grain shape and simultaneously improves rice yield and grain quality. Nat Genet 2015; 47:949–954. 26147620 10.1038/ng.3352

[bib15] Xue W, Xing Y, Weng X, et al. Natural variation in Ghd7 is an important regulator of heading date and yield potential in rice. Nat Genet 2008; 40:761–767. 18454147 10.1038/ng.143

[bib16] Fujita D, Trijatmiko KR, Tagle AG, et al. NAL1 allele from a rice landrace greatly increases yield in modern indica cultivars. Proc Natl Acad Sci USA 2013; 110:20431–20436. 24297875 10.1073/pnas.1310790110 PMC3870739

[bib17] Jiao Y, Wang Y, Xue D, et al. Regulation of OsSPL14 by OsmiR156 defines ideal plant architecture in rice. Nat Genet 2010; 42:541–544. 20495565 10.1038/ng.591

[bib18] Miura K, Ikeda M, Matsubara A, et al. OsSPL14 promotes panicle branching and higher grain productivity in rice. Nat Genet 2010; 42:545–549. 20495564 10.1038/ng.592

[bib19] Xu H, Liu Q, Yao T, Fu X. Shedding light on integrative GA signaling. Curr Opin Plant Biol 2014; 21:89–95. 25061896 10.1016/j.pbi.2014.06.010

[bib20] Nakada S, Tai I, Panier S, et al. Non-canonical inhibition of DNA damage-dependent ubiquitination by OTUB1. Nature 2010; 466:941–946. 20725033 10.1038/nature09297

[bib21] Wiener R, Zhang X, Wang T, Wolberger C. The mechanism of OTUB1-mediated inhibition of ubiquitination. Nature 2012; 483:618–622. 22367539 10.1038/nature10911 PMC3319311

[bib22] Sun XX, Challagundla KB, Dai MS. Positive regulation of p53 stability and activity by the deubiquitinating enzyme Otubain 1. EMBO J 2012; 31:576–592. 22124327 10.1038/emboj.2011.434 PMC3273389

[bib23] Herhaus L, Al-Salihi M, Macartney T, Weidlich S, Sapkota GP. OTUB1 enhances TGFbeta signalling by inhibiting the ubiquitylation and degradation of active SMAD2/3. Nat Commun 2013; 4:2519. 24071738 10.1038/ncomms3519 PMC3791481

[bib24] Ma X, Zhang Q, Zhu Q, et al. A robust CRISPR/Cas9 system for convenient, high-efficiency multiplex genome editing in monocot and dicot plants. Mol Plant 2015; 8:1274–1284. 25917172 10.1016/j.molp.2015.04.007

[bib25] Sun H, Qian Q, Wu K, et al. Heterotrimeric G proteins regulate nitrogen-use efficiency in rice. Nat Genet 2014; 46:652–656. 24777451 10.1038/ng.2958

[bib26] Liu Q, Chen X, Wu K, Fu X. Nitrogen signaling and use efficiency in plants: what's new? Curr Opin Plant Biol 2015; 27:192–198. 26340108 10.1016/j.pbi.2015.08.002

[bib27] Edelmann MJ, Iphofer A, Akutsu M, et al. Structural basis and specificity of human otubain 1-mediated deubiquitination. Biochem J 2009; 418:379–390. 18954305 10.1042/BJ20081318

[bib28] Radjacommare R, Usharani R, Kuo CH, Fu H. Distinct phylogenetic relationships and biochemical properties of *Arabidopsis* ovarian tumor-related deubiquitinases support their functional differentiation. Front Plant Sci 2014; 5:84. 24659992 10.3389/fpls.2014.00084 PMC3950621

[bib29] Wang L, Sun S, Jin J, et al. Coordinated regulation of vegetative and reproductive branching in rice. Proc Natl Acad Sci USA 2015; 112:15504–15509. 26631749 10.1073/pnas.1521949112 PMC4687603

[bib30] Si L, Chen J, Huang X, et al. OsSPL13 controls grain size in cultivated rice. Nat Genet 2016; 48:447–456. 26950093 10.1038/ng.3518

[bib31] Liu Q, Harberd NP, Fu X. SQUAMOSA promoter binding protein-like transcription factors: targets for improving cereal grain yield. Mol Plant 2016; 9:765–767. 27108382 10.1016/j.molp.2016.04.008

[bib32] Lu Z, Yu H, Xiong G, et al. Genome-wide binding analysis of the transcription activator IDEAL PLANT ARCHITECTURE1 reveals a complex network regulating rice plant architecture. Plant Cell 2013; 25:3743–3759. 24170127 10.1105/tpc.113.113639 PMC3877814

[bib33] Wang J, Yu H, Xiong G, et al. Tissue-specific ubiquitination by IPA1 INTERACTING PROTEIN 1 modulates IPA1 protein levels to regulate plant architecture in rice. Plant Cell 2017; 29:697–707. 28298520 10.1105/tpc.16.00879 PMC5435429

[bib34] Nathan JA, Kim HT, Ting L, Gygi SP, Goldberg AL. Why do cellular proteins linked to K63-polyubiquitin chains not associate with proteasomes? EMBO J 2013; 32:552–565. 23314748 10.1038/emboj.2012.354 PMC3579138

[bib35] Wang JW, Czech B, Weigel D. miR156-regulated SPL transcription factors define an endogenous flowering pathway in *Arabidopsis thaliana*. Cell 2009; 138:738–749. 19703399 10.1016/j.cell.2009.06.014

[bib36] Wu G, Park MY, Conway SR, et al. The sequential action of miR156 and miR172 regulates developmental timing in *Arabidopsis*. Cell 2009; 138:750–759. 19703400 10.1016/j.cell.2009.06.031 PMC2732587

[bib37] Jung JH, Lee HJ, Ryu JY, Park CM. SPL3/4/5 integrate developmental aging and photoperiodic signals into the FT-FD module in *Arabidopsis* flowering. Mol Plant 2016; 9:1647–1659. 27815142 10.1016/j.molp.2016.10.014

[bib38] Zhao M, Liu B, Wu K, et al. Regulation of OsmiR156h through alternative polyadenylation improves grain yield in rice. PLoS One 2015; 10:e0126154. 25954944 10.1371/journal.pone.0126154 PMC4425700

[bib39] Bracha-Drori K, Shichrur K, Katz A, et al. Detection of protein-protein interactions in plants using bimolecular fluorescence complementation. Plant J 2004; 40:419–427. 15469499 10.1111/j.1365-313X.2004.02206.x

[bib40] Wang F, Zhu D, Huang X, et al. Biochemical insights on degradation of *Arabidopsis* DELLA proteins gained from a cell-free assay system. Plant Cell 2009; 21:2378–2390. 19717618 10.1105/tpc.108.065433 PMC2751948

[bib41] Liu Z, Wu Y, Yang F, et al. BIK1 interacts with PEPRs to mediate ethylene-induced immunity. Proc Natl Acad Sci USA 2013; 110:6205–6210. 23431184 10.1073/pnas.1215543110 PMC3625333

