# Supplementary material for: Non-canonical regulation of SPL transcription factors by a human OTUB1-like deubiquitinase defines a new plant type rice associated with higher grain yield
Source: Cell Res. 2017 Aug 4;27(9):1142–56. doi: 10.1038/cr.2017.98 (PMC5587855; doi:10.1038/cr.2017.98)
Supplement: Supplementary information, Figure S1 — Protein sequence alignment. [file cr201798x1.pdf]

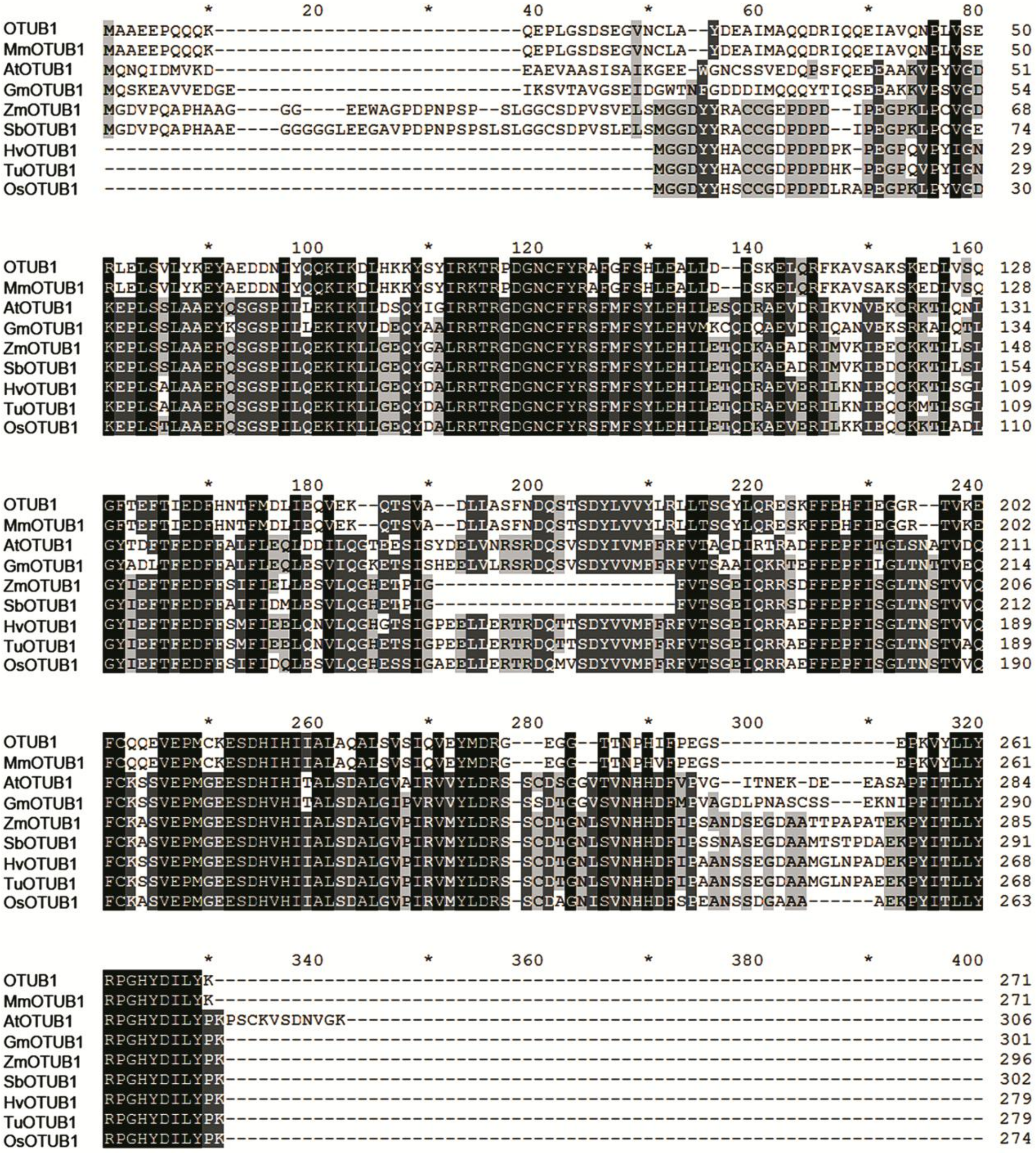

* 20 * 40 * 60 * 80
OTUB1 MAAEEPQQQK--------------------------QEPLGSDSEGVNCLA--YDEAIMAQQDRIQQEIAVQNPLVSE 50
MmOTUB1 MAAEEPQQQK--------------------------QEPLGSDSEGVNCLA--YDEAIMAQQDRIQQEIAVQNPLVSE 50
AtOTUB1 MQNQIDMVKD--------------------------EAEVAASISAIKGEE-WGNCSSVEDQPSFQEEEAAKVPYVGD 51
GmOTUB1 MQSKEAVVEDGE------------------------IKSVTAVGSEIDGWTNEGDDDIMQQQYTIQSEEAKKVPSVGD 54
ZmOTUB1 MGDVPQAPHAAG----GG----EEWAGPDDNPSP--SLGGCSDPVSVELSMGGDYYRACCGEPDPD--IPEGPKLPCVGD 68
SbOTUB1 MGDVPQAPHAAE----GGGGGLEEGAVPDPNPSPSLSLGGCSDPVSLELSMGGDYYRACCGDPDPD--IPEGPKLPCVGE 74
HvOTUB1 --MGGDYYHACCGDPDPDPK-PEGPQVPYIGN 29
TuOTUB1 --MGGDYYHACCGDPDPDHK-PEGPQVPYIGN 29
OsOTUB1 --MGGDYYHSCCGDPDPDLRAPEGPKLPYVGD 30
* 100 * 120 * 140 * 160
OTUB1 RLELSVLYKEYAEDDNIYQQKIKDLHKKYSYIRKTRPDGNCFYRAFGFSHLEALLD--DSKELQRFKAVSAKSKEDLVSQ 128
MmOTUB1 RLELSVLYKEYAEDDNIYQQKIKDLHKKYSYIRKTRPDGNCFYRAFGFSHLEALLD--DSKELQRFKAVSAKSKEDLVSQ 128
AtOTUB1 KEPLSSLAAEYQSGSPILLEKIKILDSQYIGIRRTRGDGNCFFRSFMFSYLEHILESQDRAEVDRIKVNVEKCRKTLQNL 131
GmOTUB1 KEPLSSLAAEYKSGSPILLEKIKVLDEQYAAIRRTRGDGNCFFRSFMFSYLEHVMKCQDQAEVDRIQANVEKSRKALQTL 134
ZmOTUB1 KEPLSSLAAEFQSGSPILQEKIKLLGEQYGALRRTRGDGNCFYRSFMFSYLEHILETQDKAEADRIMVKIEDCKKTLLSL 148
SbOTUB1 KEPLSSLAAEFQSGSPILQEKIKLLGEQYGALRRTRGDGNCFYRSFMFSYLEHILETQDKAEADRIMVKIEDCKKTLLSL 154
HvOTUB1 KEPLSALAAEFQSGSPILQEKIKLLGEQYDALRRTRGDGNCFYRSFMFSYLEHILETQDRAEVERILKNIEQCKKTLSGL 109
TuOTUB1 KEPLSALAAEFQSGSPILQEKIKLLGEQYDALRRTRGDGNCFYRSFMFSYLEHILETQDRAEVERILKNIEQCKMTLSGL 109
OsOTUB1 KEPLSTLAAEFQSGSPILQEKIKLLGEQYDALRRTRGDGNCFYRSFMFSYLEHILETQDKAEVERILKKIEQCKKTLADL 110
* 180 * 200 * 220 * 240
OTUB1 GFTEFTIEDFHNTFMDLIEQVEK--QTSVA--DLLASFNDQSTSDYLVVYLRLLTSGYLQRESKFFEHFIEGGR--TVKE 202
MmOTUB1 GFTEFTIEDFHNTFMDLIEQVEK--QTSVA--DLLASFNDQSTSDYLVVYLRLLTSGYLQRESKFFEHFIEGGR--TVKE 202
AtOTUB1 GYTDFTFEDFFALFLEQLDDILQGTEESISYDELVNRSRDQSVSDYIVMFFRFVTAGDIRTRADFFEPFITGLSNATVDQ 211
GmOTUB1 GYADLTFEDFFALFLEQLESVIQGKETSISHEELVLRSRDQSVSDYVVMFFRFVTSAAIQKRTEFFEPFILGLTNTTVEQ 214
ZmOTUB1 GYIEFTFEDFFSIFIELLESVLQGHETPIG---------------------FVTSGEIQRRSDFFEPFISGLTNSTVVQ 206
SbOTUB1 GYIEFTFEDFFAIFIDMLESVLQGHETPIG---------------------FVTSGEIQRRSDFFEPFISGLTNSTVVQ 212
HvOTUB1 GYIEFTFEDFFSMFIEELQNVLQGHGTSIGPEELIFERTRDQTTSDYVVMFFRFVTSGEIQRRAEFFEPFISGLTNSTVVQ 189
TuOTUB1 GYIEFTFEDFFSMFIEELQNVLQGHETSIGPEELIFERTRDQTTSDYVVMFFRFVTSGEIQRRAEFFEPFISGLTNSTVAQ 189
OsOTUB1 GYIEFTFEDFFSIFIDQLESVLQGHESSIGAEELIFERTRDQMVSDYVVMFFRFVTSGEIQRRAEFFEPFISGLTNSTVVQ 190
* 260 * 280 * 300 * 320
OTUB1 FCQQEVEPMCKESDHIHIIALAQALSVSIQVEYMDRG---EGG--TTNPHIFPEGS---------------EPKVYLLY 261
MmOTUB1 FCQQEVEPMCKESDHIHIIALAQALSVSIQVEYMDRG---EGG--TTNPHVFPEGS---------------EPKVYLLY 261
AtOTUB1 FCKSSVEPMGEESDHIHITALSDALGVAIRVVYLDRS-SCDSGGVTVNHHDFVPVG--ITNEK-DE---EASAPFITLLY 284
GmOTUB1 FCKSSVEPMGEESDHVHITALSDALGIPVRVVYLDRS-SSDTGGVSVNHHDFMPVAGDLPNASCSS---EKNIPFITLLY 290
ZmOTUB1 FCKASVEPMGEESDHVHIIALSDALGVPIRVMYLDRS-SCDTGNLSVNHHDFIPSANDSEGDAATTPAPATEKPYITLLY 285
SbOTUB1 FCKASVEPMGEESDHVHIIALSDALGVPIRVMYLDRS-SCDTGNLSVNHHDFIPSSNASEGDAAMTSTPDAEKPYITLLY 291
HvOTUB1 FCKSSVEPMGEESDHVHIIALSDALGVPIRVMYLDRS-SCDTGNLSVNHHDFIPAANSSEGDAAMGLNPADEKPYITLLY 268
TuOTUB1 FCKSSVEPMGEESDHVHIIALSDALGVPIRVMYLDRS-SCDTGNLSVNHHDFIPAANSSEGDAAMGLNPAEEKPYITLLY 268
OsOTUB1 FCKASVEPMGEESDHVHIIALSDALGVPIRVMYLDRS-SCDAGNISVNHHDFSPEANSSDGAAA------AEKPYITLLY 263
* 340 * 360 * 380 * 400
OTUB1 RPGHYDILYK-- 271
MmOTUB1 RPGHYDILYK-- 271
AtOTUB1 RPGHYDILYPKPSCKVSDNVGK-- 306
GmOTUB1 RPGHYDILYPK--- 301
ZmOTUB1 RPGHYDILYPK--- 296
SbOTUB1 RPGHYDILYPK--- 302
HvOTUB1 RPGHYDILYPK--- 279
TuOTUB1 RPGHYDILYPK--- 279
OsOTUB1 RPGHYDILYPK--- 274

Supplementary information, Figure S1. Protein sequence alignment. The sequences of human OTUB1 and its orthologues in mouse (MmOTUB1), *Arabidopsis thaliana* (AtOTUB1), soybean (GmOTUB1), maize (ZmOTUB1), sorghum (SbOTUB1), barley (HvOTUB1), wild einkorn wheat (TuOTUB1) and rice (OsOTUB1) were obtained from www.ncbi.nlm.nih.gov. The numbers on the right indicate the positions of the residues within each protein. Identical residues are indicated by dark shading, conserved residues by light shading and variable residues are not shaded.
