# Supplementary material for: Non-canonical regulation of SPL transcription factors by a human OTUB1-like deubiquitinase defines a new plant type rice associated with higher grain yield
Source: Cell Res. 2017 Aug 4;27(9):1142–56. doi: 10.1038/cr.2017.98 (PMC5587855; doi:10.1038/cr.2017.98)
Supplement: Supplementary information, Figure S2 — Analysis of pOsOTUB1::GUS expression. [file cr201798x2.pdf]

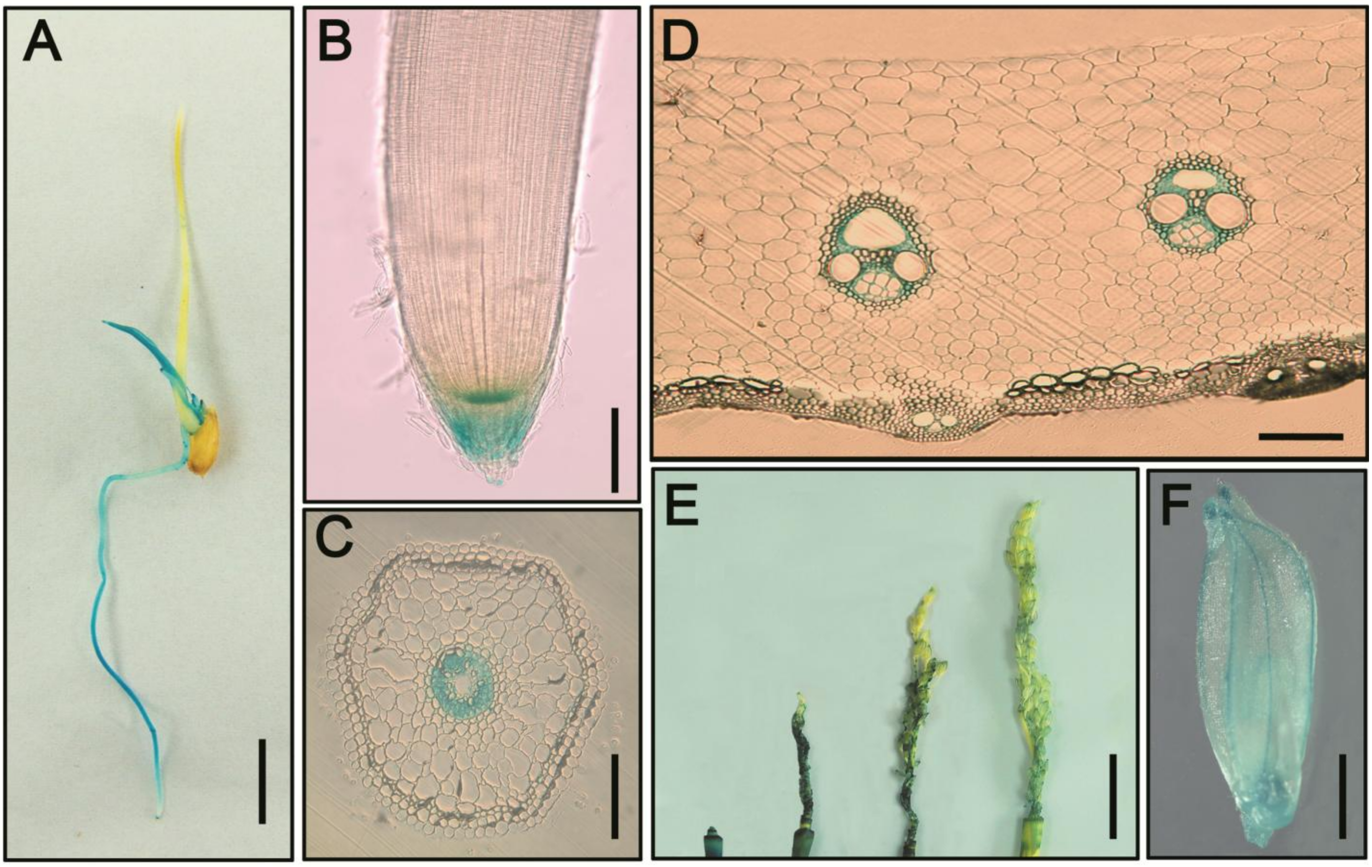

A
B
C
D
E
F

Supplementary information, Figure S2. Analysis of *pOsOTUB1::GUS* expression. **(A)** GUS expression in five-day-old seedlings. Scale bar: 1 cm. **(B)** Root tips of the plants shown in **(A)**. Scale bar: 200 μm. **(C)** Cross-section of the GUS-stained root elongation zone shown in **a**. Scale bar: 200 μm. **(D)** Cross-section of the culm. Scale bar: 100 μm. **(E)** Various stages of the panicle development. Scale bar: 1 cm. (**F**) Spikelet hull before fertilization. Scale bar: 1 mm.
