# Supplementary material for: Non-canonical regulation of SPL transcription factors by a human OTUB1-like deubiquitinase defines a new plant type rice associated with higher grain yield
Source: Cell Res. 2017 Aug 4;27(9):1142–56. doi: 10.1038/cr.2017.98 (PMC5587855; doi:10.1038/cr.2017.98)
Supplement: Supplementary information, Figure S3 — Subcellular localization of OsOTUB1.1-GFP. [file cr201798x3.pdf]

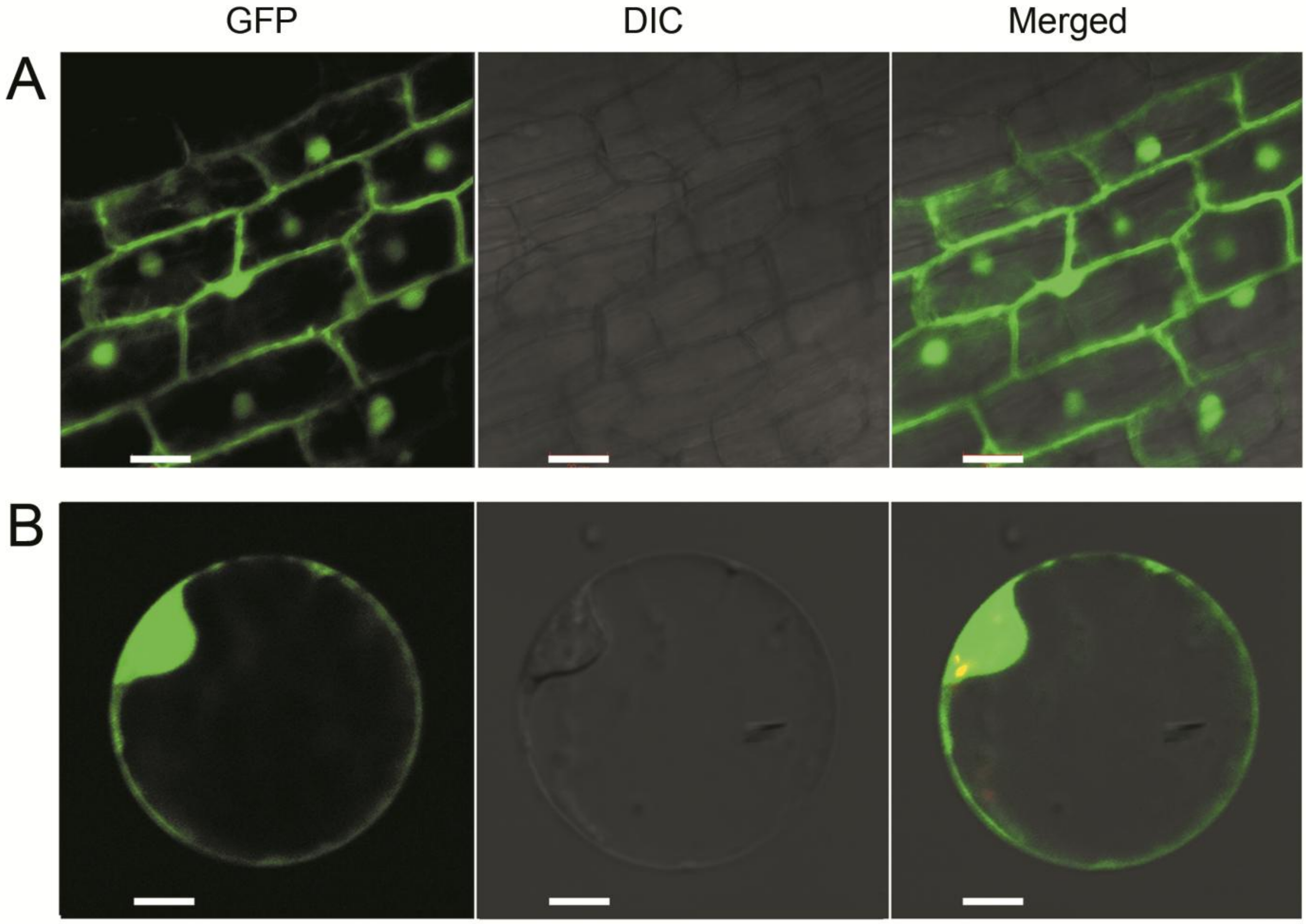
GFP
DIC
Merged
A
B

Supplementary information, Figure S3. Subcellular localization of OsOTUB1.1-GFP. **(A)** Expression of *OsOTUB1.1-GFP* in the root elongation zone. Scale bar: 20 μm. **(B)** *GFP* expression in protoplasts isolated from the leaf sheath of ZH11 plants over-expressing *OsOTUB1.1-GFP*. Scale bar: 10 μm. Panels (from left to right): GFP signal, differential interference contrast (DIC) image, merged channels.
