# Supplementary material for: Non-canonical regulation of SPL transcription factors by a human OTUB1-like deubiquitinase defines a new plant type rice associated with higher grain yield
Source: Cell Res. 2017 Aug 4;27(9):1142–56. doi: 10.1038/cr.2017.98 (PMC5587855; doi:10.1038/cr.2017.98)
Supplement: Supplementary information, Figure S4 — Effects of expressing human OTUB1 or its orthologues on ZH11-npt1 plant architecture. [file cr201798x4.pdf]

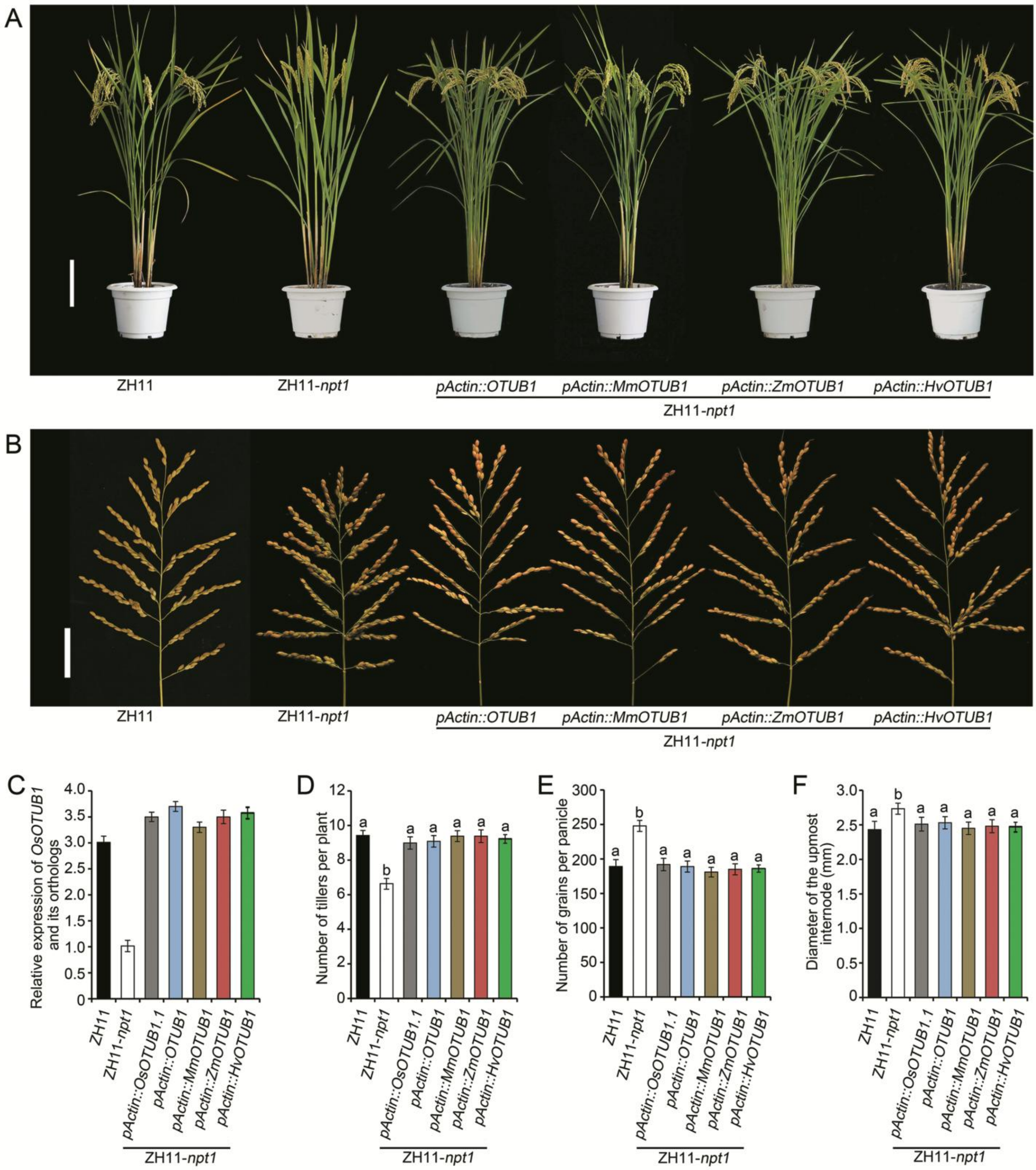
A
ZH11
ZH11-npt1
pActin::OTUB1
pActin::MmOTUB1
pActin::ZmOTUB1
pActin::HvOTUB1
ZH11-npt1
B
ZH11
ZH11-npt1
pActin::OTUB1
pActin::MmOTUB1
pActin::ZmOTUB1
pActin::HvOTUB1
ZH11-npt1
C
Relative expression of OsOTUB1 and its orthologs
D
Number of tillers per plant
E
Number of grains per panicle
F
Diameter of the upmost internode (mm)
ZH11
ZH11-npt1
pActin::OsOTUB1.1
pActin::OTUB1
pActin::MmOTUB1
pActin::ZmOTUB1
pActin::HvOTUB1
ZH11-npt1

Supplementary information, Figure S4. Effects of expressing human *OTUB1* or its orthologues on ZH11-*npt1* plant architecture. **(A)** Plant morphology. Scale bar: 20 cm. **(B)** Panicle morphology. Scale bar: 5 cm. **(C)** Abundance of the transcripts of *OTUB1* (or its orthologues) in young panicles relative to the level of *OsOTUB1* in ZH11-*npt1* plants. Data are shown as the mean ± s.e.m. (n = 3). **(D)** Number of tillers per plant. **(E)** Number of grains per panicle. **(F)** Diameter of the uppermost internode. Data are shown as the mean ± s.e.m. (n = 30). All phenotypic data were measured in paddy-grown rice plants under normal cultivation conditions. The presence of the same lowercase letter denotes a non-significant difference between means ($P < 0.05$, panels **D** to **F**).
