# Supplementary material for: Non-canonical regulation of SPL transcription factors by a human OTUB1-like deubiquitinase defines a new plant type rice associated with higher grain yield
Source: Cell Res. 2017 Aug 4;27(9):1142–56. doi: 10.1038/cr.2017.98 (PMC5587855; doi:10.1038/cr.2017.98)
Supplement: Supplementary information, Figure S5 — The SBP domain is required for the OsSPL14-OsOTUB1 interaction. [file cr201798x5.pdf]

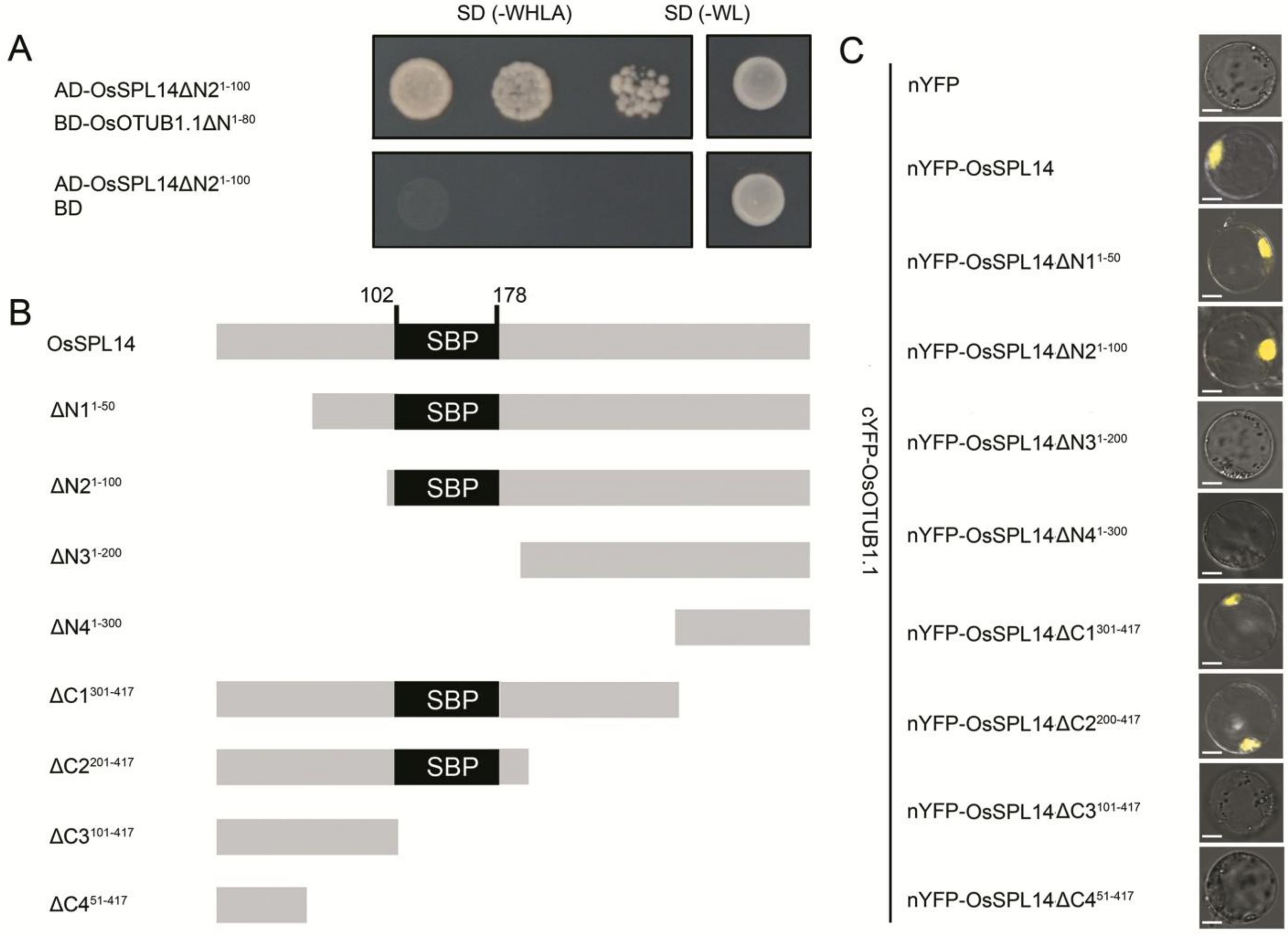
SD (-WHLA)
SD (-WL)
A
AD-OsSPL14ΔN2 1-100
BD-OsOTUB1.1ΔN 1-80
AD-OsSPL14ΔN2 1-100
BD
B
102
178
OsSPL14
SBP
ΔN1 1-50
ΔN2 1-100
ΔN3 1-200
ΔN4 1-300
ΔC1 301-417
ΔC2 201-417
ΔC3 101-417
ΔC4 51-417
C
cYFP-OsOTUB1.1
nYFP
nYFP-OsSPL14
nYFP-OsSPL14ΔN1 1-50
nYFP-OsSPL14ΔN2 1-100
nYFP-OsSPL14ΔN3 1-200
nYFP-OsSPL14ΔN4 1-300
nYFP-OsSPL14ΔC1 301-417
nYFP-OsSPL14ΔC2 200-417
nYFP-OsSPL14ΔC3 101-417
nYFP-OsSPL14ΔC4 51-417

Supplementary information, Figure S5. The SBP domain is required for the OsSPL14-OsOTUB1 interaction. **(A)** Yeast two-hybrid assays confirm the interaction between the C-termini of OsOTUB1 and OsSPL14. **(B)** Schematic representation of the deleted and non-deleted versions of the OsSPL14 protein used for the BiFC assays. **(C)** BiFC assays. Scale bar: 10 μm.
