# Supplementary material for: Non-canonical regulation of SPL transcription factors by a human OTUB1-like deubiquitinase defines a new plant type rice associated with higher grain yield
Source: Cell Res. 2017 Aug 4;27(9):1142–56. doi: 10.1038/cr.2017.98 (PMC5587855; doi:10.1038/cr.2017.98)
Supplement: Supplementary information, Figure S6 — OsOTUB1 interacts with rice SPL transcription factors. [file cr201798x6.pdf]

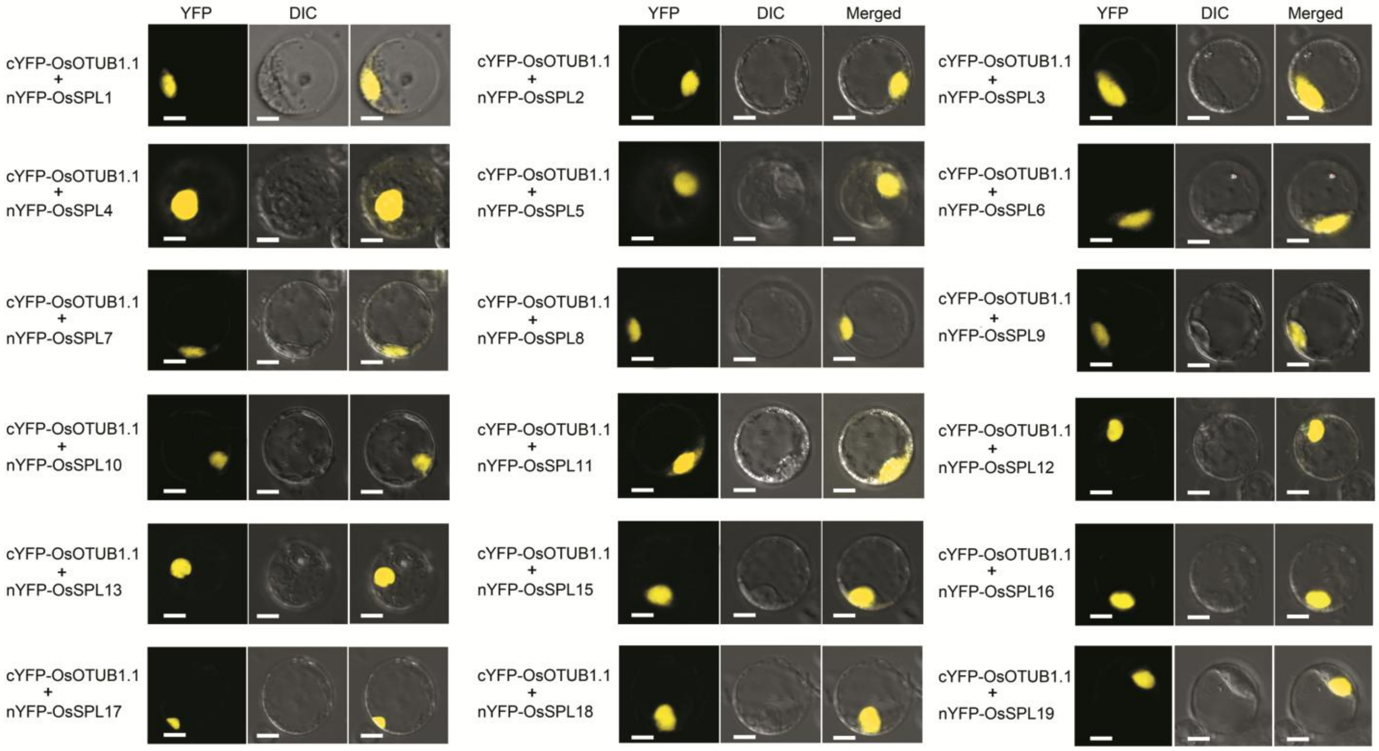
YFP
DIC
Merged
cYFP-OsOTUB1.1 + nYFP-OsSPL1
cYFP-OsOTUB1.1 + nYFP-OsSPL2
cYFP-OsOTUB1.1 + nYFP-OsSPL3
cYFP-OsOTUB1.1 + nYFP-OsSPL4
cYFP-OsOTUB1.1 + nYFP-OsSPL5
cYFP-OsOTUB1.1 + nYFP-OsSPL6
cYFP-OsOTUB1.1 + nYFP-OsSPL7
cYFP-OsOTUB1.1 + nYFP-OsSPL8
cYFP-OsOTUB1.1 + nYFP-OsSPL9
cYFP-OsOTUB1.1 + nYFP-OsSPL10
cYFP-OsOTUB1.1 + nYFP-OsSPL11
cYFP-OsOTUB1.1 + nYFP-OsSPL12
cYFP-OsOTUB1.1 + nYFP-OsSPL13
cYFP-OsOTUB1.1 + nYFP-OsSPL15
cYFP-OsOTUB1.1 + nYFP-OsSPL16
cYFP-OsOTUB1.1 + nYFP-OsSPL17
cYFP-OsOTUB1.1 + nYFP-OsSPL18
cYFP-OsOTUB1.1 + nYFP-OsSPL19

Supplementary information, Figure S6 OsOTUB1 interacts with rice SPL transcription factors. BiFC assays were performed using rice protoplasts; the C-terminus of YFP-tagged OsOTUB1.1 was co-transformed with the N-terminus of YFP-tagged OsSPLs. Panels (from left to right): YFP signal, differential interference contrast (DIC) image, merged channel. Scale bar: 10 μm.
