# Supplementary material for: Non-canonical regulation of SPL transcription factors by a human OTUB1-like deubiquitinase defines a new plant type rice associated with higher grain yield
Source: Cell Res. 2017 Aug 4;27(9):1142–56. doi: 10.1038/cr.2017.98 (PMC5587855; doi:10.1038/cr.2017.98)
Supplement: Supplementary information, Figure S7 — Effect of the OsSPL14-OsOTUB1 interaction on the DNA binding affinity of OsSPL14. [file cr201798x7.pdf]

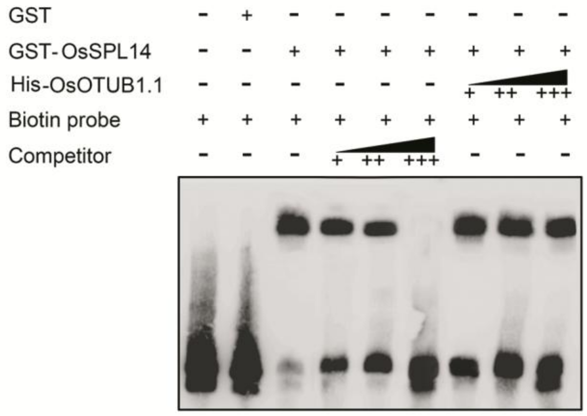
GST
GST-OsSPL14
His-OsOTUB1.1
Biotin probe
Competitor

Supplementary information, Figure S7 Effect of the OsSPL14-OsOTUB1 interaction on the DNA binding affinity of OsSPL14. Competition for OsSPL14-GST protein binding was performed with unlabelled probes (at 10×, 20× or 50×) containing the GTAC-box motifs from the promoter of the *DEP1* gene, or with 1×, 2× or 4× unlabelled His-OsOTUB1.1 fusion protein.
