# Supplementary material for: Non-canonical regulation of SPL transcription factors by a human OTUB1-like deubiquitinase defines a new plant type rice associated with higher grain yield
Source: Cell Res. 2017 Aug 4;27(9):1142–56. doi: 10.1038/cr.2017.98 (PMC5587855; doi:10.1038/cr.2017.98)
Supplement: Supplementary information, Table S1 — Identification of the putative OsOTUB1-interacting proteins using yeast two-hybrid assays. [file cr201798x8.pdf]

**Supplementary information, Table S1.**

Identification of the putative OsOTUB1-interacting proteins using yeast two-hybrid assays.

| Bait | Gene ID | Screening frequency | CDS length of each clone (bps) | Gene Product |
|---|---|---|---|---|
| Full-length OsOTUB1 | LOC_Os01g06060 | 1 | 483 | gibberellin receptor GID1L2 |
| | LOC_Os01g07520 | 1 | 1389 | proline-rich family protein |
| | LOC_Os01g46926 | 1 | 441 | ubiquitin-conjugating enzyme |
| | LOC_Os01g56200 | 1 | 1530 | BTBA2 - Bric-a-Brac, Tramtrack |
| | LOC_Os01g60410 | 1 | 447 | ubiquitin-conjugating enzyme |
| | LOC_Os01g67134 | 2 | 543，687 | ribosomal L18p/L5e family protein |
| | LOC_Os01g70140 | 1 | 585 | ubiquitin-conjugating enzyme E2-22 kDa |
| | LOC_Os02g01740 | 1 | 321 | U5 small nuclear ribonucleoprotein 200 kDa helicase |
| | LOC_Os02g08380 | 1 | 465 | CR084 protein |
| | LOC_Os02g11050 | 1 | 942 | 26S protease regulatory subunit |
| | LOC_Os02g16040 | 1 | 447 | ubiquitin-conjugating enzyme |
| | LOC_Os02g49720 | 1 | 825 | aldehyde dehydrogenase |
| | LOC_Os03g30430 | 1 | 498 | nitrilase-associated protein |
| | LOC_Os03g53710 | 1 | 1119 | aldose 1-epimerase |
| | LOC_Os04g57220 | 1 | 447 | ubiquitin-conjugating enzyme |
| | LOC_Os05g02070 | 1 | 255 | expressed protein |
| | LOC_Os05g03550 | 1 | 1149 | MYB family transcription factor |
| | LOC_Os05g12680 | 1 | 1170 | retrotransposon protein, putative, unclassified |
| | LOC_Os05g27820 | 1 | 285 | patellin protein |
| | LOC_Os05g34050 | 1 | 729 | bZIP transcription factor domain containing protein |
| | LOC_Os05g34070 | 1 | 630 | DIP1 |

| | | | | |
|---|---|---|---|---|
| Full-length OsOTUB1 | LOC_Os05g36280 | 1 | 411 | histone H3 |
| | LOC_Os05g43576 | 1 | 957 | radical SAM enzyme |
| | LOC_Os05g45950 | 1 | 843 | outer mitochondrial membrane porin |
| | LOC_Os06g01210 | 1 | 465 | plastocyanin, chloroplast precursor |
| | LOC_Os06g30970 | 1 | 447 | ubiquitin-conjugating enzyme |
| | LOC_Os06g37560 | 1 | 1224 | beta-galactosidase precursor |
| | LOC_Os06g43770 | 1 | 714 | expressed protein |
| | LOC_Os07g04840 | 1 | 765 | PsbP |
| | LOC_Os09g17650 | 1 | 579 | expressed protein |
| | LOC_Os09g19734 | 2 | 1431，2442 | isochorismate synthase 1, chloroplast precursor |
| | LOC_Os09g27030 | 1 | 975 | oryzain gamma chain precursor |
| | LOC_Os09g39590 | 1 | 1029 | metallo-beta-lactamase |
| | LOC_Os10g42439 | 1 | 954 | heat shock protein DnaJ |
| | LOC_Os11g08300 | 1 | 1353 | aldehyde dehydrogenase |
| | LOC_Os12g03070 | 1 | 513 | FHA domain containing protein |
| | LOC_Os12g44000 | 1 | 483 | ubiquitin-conjugating enzyme E2 |

| | | | | |
|---|---|---|---|---|
| C-terminus of OsOTUB1 | LOC_Os03g47610 | 1 | 1503 | thiamine biosynthesis protein thiC |
| | LOC_Os04g30760 | 1 | 318 | 3-oxoacyl-reductase, chloroplast precursor |
| | LOC_Os04g33300 | 1 | 300 | amino acid kinase |
| | LOC_Os04g53540 | 1 | 784 | homeobox and START domains containing protein |
| | LOC_Os06g11610 | 1 | 248 | heat shock 22 kDa protein, mitochondrial precursor |
| | LOC_Os06g50900 | 1 | 213 | expressed protein |
| | LOC_Os07g42450 | 1 | 141 | ribosomal protein S2 |
| | LOC_Os07g44610 | 1 | 660 | pyrrolidone-carboxylate peptidase |
| | LOC_Os07g46440 | 1 | 345 | ribosomal protein |
| | LOC_Os07g47710 | 1 | 135 | 60S ribosomal protein L22-2 |
| | LOC_Os07g48080 | 1 | 519 | expressed protein |
| | LOC_Os08g37050 | 1 | 663 | gibberellin receptor GID1L2 |
| | LOC_Os08g38780 | 1 | 315 | core histone H2A/H2B/H3/H4 |
| | LOC_Os08g39890 | 1 | 951 | OsSPL14, SBP-box gene family member |
| | LOC_Os09g20460 | 1 | 438 | VQ domain containing protein |
| | LOC_Os10g32550 | 1 | 609 | T-complex protein |
| | LOC_Os10g35030 | 1 | 906 | IAP100 |
| | LOC_Os10g35630 | 1 | 385 | cystathionin beta synthase protein |
| | LOC_Os10g37740 | 1 | 222 | CGMC_GSK.9 - CGMC includes CDA, MAPK, GSK3, and CLKC kinases |
| | LOC_Os10g40360 | 1 | 639 | proline oxidase, mitochondrial precursor |
| | LOC_Os10g40720 | 1 | 402 | expansin precursor |
| | LOC_Os10g41410 | 1 | 174 | nucleoside diphosphate kinase |
| | LOC_Os11g04950 | 1 | 318 | splicing factor |
| | LOC_Os11g05130 | 1 | 679 | PHD-finger family protein |
| | LOC_Os12g19381 | 1 | 279 | ribulose bisphosphate carboxylase small chain |
