# Supplementary material for: Non-canonical regulation of SPL transcription factors by a human OTUB1-like deubiquitinase defines a new plant type rice associated with higher grain yield
Source: Cell Res. 2017 Aug 4;27(9):1142–56. doi: 10.1038/cr.2017.98 (PMC5587855; doi:10.1038/cr.2017.98)
Supplement: Supplementary information, Table S2 — The common target genes regulated by npt1 and OsSPL14WFP alleles. [file cr201798x9.pdf]

**Supplementary information, Table S2.**

The common target genes regulated by *npt1* and *OsSPL14*$^{WFP}$ alleles.

| Gene ID | Description |
|---|---|
| LOC_Os01g04800 | B3 DNA binding domain containing protein |
| LOC_Os01g06160 | retrotransposon protein, putative, Ty3-gypsy subclass |
| LOC_Os01g07530 | uncharacterized glycosyltransferase |
| LOC_Os01g09320 | NADP-dependent malic enzyme, chloroplast precursor |
| LOC_Os01g09640 | Myb transcription factor |
| LOC_Os01g10250 | hydrolase, alpha/beta fold family domain containing protein |
| LOC_Os01g14590 | pathogen-related protein |
| LOC_Os01g15520 | expressed protein |
| LOC_Os01g15934 | retrotransposon protein, putative, unclassified |
| LOC_Os01g17396 | expressed protein |
| LOC_Os01g19770 | mitochondrial import inner membrane translocase subunit Tim17 |
| LOC_Os01g19800 | zinc finger, C3HC4 type |
| LOC_Os01g19940 | expressed protein |
| LOC_Os01g20910 | zinc finger, C3HC4 type domain containing protein |
| LOC_Os01g21250 | late embryogenesis abundant protein |
| LOC_Os01g24820 | NB-ARC domain containing protein |
| LOC_Os01g29650 | retrotransposon protein, putative, Ty1-copia subclass |
| LOC_Os01g31040 | retrotransposon protein, putative, unclassified |
| LOC_Os01g34790 | expressed protein |
| LOC_Os01g36010 | retrotransposon protein, putative, Ty3-gypsy subclass |
| LOC_Os01g38580 | beta-carotene 9,10-dioxygenase |

| | |
|---|---|
| LOC_Os01g39070 | DUF260 domain containing protein, putative |
| LOC_Os01g41260 | OsFBD2 - F-box and FBD domain containing protein |
| LOC_Os01g41834 | chalcone synthase |
| LOC_Os01g42370 | pleiotropic drug resistance protein |
| LOC_Os01g43480 | AAA-type ATPase family protein |
| LOC_Os01g43590 | HSF-type DNA-binding domain containing protein |
| LOC_Os01g45250 | DUF1645 domain containing protein |
| LOC_Os01g45659 | expressed protein |
| LOC_Os01g45830 | sulfate transporter |
| LOC_Os01g46260 | expressed protein |
| LOC_Os01g47070 | glycosyl hydrolase |
| LOC_Os01g47262 | AMSH-like protease |
| LOC_Os01g47570 | expressed protein |
| LOC_Os01g50700 | dehydrin family protein |
| LOC_Os01g50910 | late embryogenesis abundant protein, group 3 |
| LOC_Os01g51180 | transposon protein, putative, unclassified |
| LOC_Os01g52120 | expressed protein |
| LOC_Os01g52260 | serine acetyltransferase protein |
| LOC_Os01g52650 | POEI44 - Pollen Ole e I allergen and extensin family protein precursor |
| LOC_Os01g52830 | DUF1264 domain containing protein |
| LOC_Os01g53090 | pathogen-related protein |
| LOC_Os01g53420 | anthocyanidin 5,3-O-glucosyltransferase |
| LOC_Os01g56160 | expressed protein |
| LOC_Os01g57840 | expressed protein |
| LOC_Os01g58480 | expressed protein |
| LOC_Os01g58930 | hypothetical protein |

| | |
|---|---|
| LOC_Os01g59690 | OsFBX28 - F-box domain containing protein |
| LOC_Os01g62610 | peptidyl-prolyl cis-trans isomerase |
| LOC_Os01g62750 | expressed protein |
| LOC_Os01g62760 | protein phosphatase 2C |
| LOC_Os01g64670 | soluble inorganic pyrophosphatase |
| LOC_Os01g64949 | expressed protein |
| LOC_Os01g66120 | No apical meristem protein |
| LOC_Os01g66530 | ARGOS |
| LOC_Os01g69010 | MATE efflux protein |
| LOC_Os01g72270 | cytochrome P450 |
| LOC_Os01g73100 | retrotransposon protein, putative, unclassified |
| LOC_Os01g74450 | aquaporin protein |
| LOC_Os01g74480 | cupin domain containing protein |
| LOC_Os01g74630 | expressed protein |
| LOC_Os02g03400 | microtubule associated protein |
| LOC_Os02g04780 | expressed protein |
| LOC_Os02g05210 | retrotransposon protein, putative, unclassified |
| LOC_Os02g06410 | CBS domain containing membrane protein |
| LOC_Os02g09190 | cytochrome P450 |
| LOC_Os02g09240 | cytochrome P450 71D8 |
| LOC_Os02g12310 | no apical meristem protein |
| LOC_Os02g12380 | histone deacetylase |
| LOC_Os02g12680 | cytochrome P450 |
| LOC_Os02g13370 | expressed protein |
| LOC_Os02g13800 | HSF-type DNA-binding domain containing protein |
| LOC_Os02g14970 | expressed protein |

| | |
|---|---|
| LOC_Os02g15700 | expressed protein |
| LOC_Os02g16010 | retrotransposon protein, putative, unclassified |
| LOC_Os02g26420 | retrotransposon protein, putative, unclassified |
| LOC_Os02g26470 | expressed protein |
| LOC_Os02g26480 | OsSCP7 - Putative Serine Carboxypeptidase homologue, expressed |
| LOC_Os02g27310 | TKL_IRAK_DUF26-lc.6 - DUF26 kinases have homology to DUF26 containing loci |
| LOC_Os02g29570 | retrotransposon protein, putative, Ty3-gypsy subclass |
| LOC_Os02g32840 | zinc finger A20 and AN1 domain-containing stress-associated protein |
| LOC_Os02g32950 | RCN2 Centroradialis-like1 homologous to TFL1 |
| LOC_Os02g33090 | expressed protein |
| LOC_Os02g33420 | ATPase, AAA family protein |
| LOC_Os02g35090 | expressed protein |
| LOC_Os02g36350 | expressed protein |
| LOC_Os02g37320 | heavy metal associated domain containing protein |
| LOC_Os02g37330 | heavy metal associated domain containing protein |
| LOC_Os02g37490 | glycine-rich cell wall structural protein precursor |
| LOC_Os02g39790 | CPuORF9 - conserved peptide uORF-containing transcript |
| LOC_Os02g39900 | transposon protein, putative, unclassified |
| LOC_Os02g40040 | expressed protein |
| LOC_Os02g41860 | aquaporin protein |
| LOC_Os02g43110 | sodium/calcium exchanger 1 precursor |
| LOC_Os02g43330 | homeobox associated leucine zipper |
| LOC_Os02g44090 | zinc finger protein |
| LOC_Os02g44490 | anthranilate phosphoribosyltransferase |
| LOC_Os02g44990 | OsFBDUF13 - F-box and DUF domain containing protein |
| LOC_Os02g45000 | expressed protein |

| | |
|---|---|
| LOC_Os02g46030 | MYB family transcription factor |
| LOC_Os02g47200 | transposon protein, putative, unclassified |
| LOC_Os02g48710 | expressed protein |
| LOC_Os02g49720 | aldehyde dehydrogenase |
| LOC_Os02g49860 | AWPM-19-like membrane family protein |
| LOC_Os02g51110 | aquaporin protein |
| LOC_Os02g51960 | transposon protein, putative, unclassified |
| LOC_Os02g52210 | zinc finger, C3HC4 type domain containing protein |
| LOC_Os02g52790 | PB1 domain containing protein |
| LOC_Os02g52910 | zinc finger protein |
| LOC_Os02g55400 | ATPase 8, plasma membrane-type |
| LOC_Os02g56240 | retrotransposon protein, putative, Ty3-gypsy subclass |
| LOC_Os02g58290 | expressed protein |
| LOC_Os03g02230 | expressed protein |
| LOC_Os03g02300 | expressed protein |
| LOC_Os03g02470 | expressed protein |
| LOC_Os03g04080 | expressed protein |
| LOC_Os03g05440 | DUF677 domain containing protein |
| LOC_Os03g06080 | heavy metal-associated domain containing protein |
| LOC_Os03g08930 | helix-loop-helix DNA-binding protein |
| LOC_Os03g09300 | pumilio-family RNA binding repeat containing protein |
| LOC_Os03g10300 | haemolysin-III |
| LOC_Os03g10810 | HLS, putative, expressed |
| LOC_Os03g16170 | protein phosphatase 2C, putative |
| LOC_Os03g16530 | retrotransposon protein, putative, unclassified |
| LOC_Os03g18850 | pathogenesis-related Bet v I family protein |

| | |
|---|---|
| LOC_Os03g19010 | peroxisomal biogenesis factor 11 |
| LOC_Os03g19270 | universal stress protein domain containing protein |
| LOC_Os03g20120 | glycosyl transferase 8 domain containing protein |
| LOC_Os03g20680 | late embryogenesis abundant protein 1 |
| LOC_Os03g21650 | expressed protein |
| LOC_Os03g24810 | expressed protein |
| LOC_Os03g26210 | helix-loop-helix DNA-binding domain containing protein |
| LOC_Os03g26490 | expressed protein |
| LOC_Os03g26870 | WD-40 repeat family protein |
| LOC_Os03g29140 | expressed protein |
| LOC_Os03g29970 | histone-like transcription factor and archaeal histone |
| LOC_Os03g31026 | retrotransposon protein, putative, unclassified |
| LOC_Os03g32636 | hypothetical protein |
| LOC_Os03g35640 | retrotransposon protein, putative, unclassified |
| LOC_Os03g39170 | frigida, putative, expressed |
| LOC_Os03g39400 | retrotransposon protein, putative, Ty1-copia subclass |
| LOC_Os03g41130 | expressed protein |
| LOC_Os03g42520 | expressed protein |
| LOC_Os03g42600 | expressed protein |
| LOC_Os03g43720 | transporter family protein |
| LOC_Os03g44140 | OsWAK26 - OsWAK receptor-like protein kinase |
| LOC_Os03g46100 | cupin domain containing protein |
| LOC_Os03g46200 | acetyltransferase, GNAT family |
| LOC_Os03g46980 | retrotransposon protein, putative, unclassified |
| LOC_Os03g47590 | retrotransposon protein, putative, Ty3-gypsy subclass |
| LOC_Os03g48471 | ATP synthase subunit alpha |

| | |
|---|---|
| LOC_Os03g49190 | oleosin, putative |
| LOC_Os03g49440 | phosphatase |
| LOC_Os03g49530 | transposon protein |
| LOC_Os03g49630 | expressed protein |
| LOC_Os03g49880 | TCP family transcription factor |
| LOC_Os03g50250 | DnaK family protein |
| LOC_Os03g51350 | expressed protein |
| LOC_Os03g51390 | expressed protein |
| LOC_Os03g51980 | expressed protein |
| LOC_Os03g53440 | expressed protein |
| LOC_Os03g53900 | universal stress protein domain containing protein |
| LOC_Os03g54170 | OsMADS34 - MADS-box family gene with MIKCc type-box |
| LOC_Os03g55230 | cytochrome P450, putative, expressed |
| LOC_Os03g57380 | retrotransposon protein, putative, unclassified |
| LOC_Os03g59320 | expressed protein |
| LOC_Os03g60580 | actin-depolymerizing factor |
| LOC_Os03g61150 | expressed protein |
| LOC_Os03g61160 | expressed protein |
| LOC_Os03g61360 | hydrolase, alpha/beta fold family domain containing protein |
| LOC_Os03g63870 | expressed protein |
| LOC_Os04g10060 | ent-kaurene synthase, chloroplast precursor |
| LOC_Os04g12950 | indole-3-acetate beta-glucosyltransferase |
| LOC_Os04g13890 | retrotransposon protein, putative, Ty3-gypsy subclass |
| LOC_Os04g17100 | heavy metal-associated domain containing protein |
| LOC_Os04g20749 | expressed protein |
| LOC_Os04g23600 | D-mannose binding lectin family protein |

| | |
|---|---|
| LOC_Os04g25440 | cytokinin-O-glucosyltransferase 2 |
| LOC_Os04g26310 | transposon protein, putative, CACTA, En/Spm sub-class |
| LOC_Os04g27410 | MYB family transcription facto |
| LOC_Os04g28840 | RALFL35 |
| LOC_Os04g30490 | MATE efflux family protein |
| LOC_Os04g30750 | expressed protein |
| LOC_Os04g31710 | expressed protein |
| LOC_Os04g31790 | expressed protein |
| LOC_Os04g33650 | expressed protein |
| LOC_Os04g33710 | expressed protein |
| LOC_Os04g33830 | membrane protein |
| LOC_Os04g35490 | expressed protein |
| LOC_Os04g35840 | T-complex protein 11 |
| LOC_Os04g37619 | zeaxanthin epoxidase, chloroplast precursor |
| LOC_Os04g37640 | xyloglucan fucosyltransferase |
| LOC_Os04g37680 | alpha/beta hydrolase fold |
| LOC_Os04g37740 | zinc finger, C3HC4 type domain containing protein |
| LOC_Os04g37880 | expressed protein |
| LOC_Os04g39350 | heavy metal associated domain containing protein |
| LOC_Os04g40070 | GRAM and C2 domains containing protein |
| LOC_Os04g40470 | cytochrome P450 |
| LOC_Os04g40990 | malate synthase, glyoxysomal |
| LOC_Os04g41570 | ethylene-responsive protein related |
| LOC_Os04g42399 | retrotransposon protein, putative, Ty1-copia subclass |
| LOC_Os04g42590 | expressed protein |
| LOC_Os04g43990 | DUF584 domain containing protein |

| | |
|---|---|
| LOC_Os04g44070 | expressed protein |
| LOC_Os04g45810 | homeobox associated leucine zipper |
| LOC_Os04g45900 | transposon protein, putative, unclassified |
| LOC_Os04g45920 | protein kinase domain containing protein |
| LOC_Os04g46120 | plastocyanin-like domain containing protein |
| LOC_Os04g46970 | glucosyltransferase |
| LOC_Os04g47740 | retrotransposon protein, putative, unclassified |
| LOC_Os04g48150 | retrotransposon protein, putative, unclassified |
| LOC_Os04g48360 | expressed protein |
| LOC_Os04g49748 | purine permease |
| LOC_Os04g49980 | late embryogenesis abundant group 1 |
| LOC_Os04g52110 | late embryogenesis abundant protein, group 3 |
| LOC_Os04g54310 | expressed protein |
| LOC_Os04g54980 | expressed protein |
| LOC_Os04g55000 | cullin |
| LOC_Os04g56030 | glycine-rich cell wall structural protein precursor |
| LOC_Os04g56110 | protein kinase |
| LOC_Os04g57180 | flavonol synthase/flavanone 3-hydroxylase |
| LOC_Os04g58280 | stem-specific protein TSJT1 |
| LOC_Os04g59260 | peroxidase precursor |
| LOC_Os04g59300 | strictosidine synthase |
| LOC_Os04g59540 | phosphatidylinositol-4-phosphate 5-Kinase |
| LOC_Os05g01380 | polygalacturonase inhibitor precursor |
| LOC_Os05g03040 | AP2 domain containing protein |
| LOC_Os05g03140 | tetraspanin family protein |
| LOC_Os05g04700 | OsRCI2-6 - Hydrophobic protein LTI6B |

| | |
|---|---|
| LOC_Os05g05500 | OsFBX159 - F-box domain containing protein |
| LOC_Os05g05930 | peripheral-type benzodiazepine receptor |
| LOC_Os05g05990 | transposon protein, putative, unclassified |
| LOC_Os05g06410 | expressed protein |
| LOC_Os05g07120 | basic helix-loop-helix |
| LOC_Os05g10650 | 6-phosphofructokinase |
| LOC_Os05g15530 | aminotransferase domain containing protein |
| LOC_Os05g16054 | expressed protein |
| LOC_Os05g16060 | expressed protein |
| LOC_Os05g18280 | kelch domain containing protein |
| LOC_Os05g25040 | expressed protein |
| LOC_Os05g27780 | expressed protein |
| LOC_Os05g28210 | small hydrophilic plant seed protein |
| LOC_Os05g28320 | myb-like DNA-binding domain containing protein |
| LOC_Os05g31020 | eukaryotic peptide chain release factor subunit 1-1 |
| LOC_Os05g31670 | AWPM-19-like membrane family protein |
| LOC_Os05g33120 | retrotransposon protein, putative, unclassified |
| LOC_Os05g33730 | gibberellin receptor GID1L2 |
| LOC_Os05g33840 | transketolase |
| LOC_Os05g34830 | No apical meristem protein |
| LOC_Os05g35140 | nodulin MtN3 family protein |
| LOC_Os05g37060 | MYB family transcription factor |
| LOC_Os05g37520 | expressed protein |
| LOC_Os05g38040 | expressed protein |
| LOC_Os05g38290 | protein phosphatase 2C, putative |
| LOC_Os05g39250 | phosphatidylethanolamine-binding protein |

| | |
|---|---|
| LOC_Os05g39540 | metal cation transporter |
| LOC_Os05g41370 | TKL_IRAK_DUF26-la.1 - DUF26 kinases have homology to DUF26 containing loci |
| LOC_Os05g41820 | expressed protein |
| LOC_Os05g43010 | retrotransposon protein, putative, Ty3-gypsy subclass |
| LOC_Os05g43840 | CAMK_KIN1/SNF1/Nim1_like.25 - CAMK includes calcium/calmodulin depedent protein kinases |
| LOC_Os05g45640 | retrotransposon protein, putative, unclassified |
| LOC_Os05g46460 | hydrolase, alpha/beta fold family domain containing protein, expressed |
| LOC_Os05g47950 | UDP-glucoronosyl and UDP-glucosyl transferase domain containing protein |
| LOC_Os05g48180 | expressed protein |
| LOC_Os05g48200 | glutamate synthase, chloroplast precursor |
| LOC_Os05g48416 | lipase-related |
| LOC_Os05g49420 | transcription factor, putative |
| LOC_Os05g51680 | SCP-like extracellular protein |
| LOC_Os05g51750 | aspartyl protease family protein |
| LOC_Os06g01340 | CCT/B-box zinc finger protein |
| LOC_Os06g04220 | expressed protein |
| LOC_Os06g04930 | expressed protein |
| LOC_Os06g05420 | expressed protein |
| LOC_Os06g07030 | AP2 domain containing protein |
| LOC_Os06g09960 | expressed protein |
| LOC_Os06g10380 | transposon protein, putative, CACTA, En/Spm sub-class |
| LOC_Os06g10790 | lectin-like receptor kinase |
| LOC_Os06g11840 | trehalose phosphatase |
| LOC_Os06g12710 | retrotransposon protein, putative, Ty3-gypsy subclass |
| LOC_Os06g13190 | expressed protein |
| LOC_Os06g16100 | retrotransposon, putative, centromere-specific |

| | |
|---|---|
| LOC_Os06g16900 | transposon protein, putative, CACTA, En/Spm sub-class |
| LOC_Os06g17610 | retrotransposon protein, putative, Ty3-gypsy subclass |
| LOC_Os06g20060 | expressed protein |
| LOC_Os06g21910 | late embryogenesis abundant group 1 |
| LOC_Os06g23260 | transposon protein, putative, CACTA, En/Spm sub-class |
| LOC_Os06g23350 | late embryogenesis abundant protein D-34 |
| LOC_Os06g24430 | expressed protein |
| LOC_Os06g25010 | glycosyl hydrolase |
| LOC_Os06g32890 | retrotransposon protein, putative, Ty3-gypsy subclass |
| LOC_Os06g33970 | VQ domain containing protein |
| LOC_Os06g35520 | peroxidase precursor |
| LOC_Os06g38294 | peptide transporter PTR2 |
| LOC_Os06g38603 | retrotransposon protein, putative, unclassified |
| LOC_Os06g40640 | fructose-bisphospate aldolase isozyme |
| LOC_Os06g40809 | expressed protein |
| LOC_Os06g44190 | expressed protein |
| LOC_Os06g46740 | early nodulin 20 precursor |
| LOC_Os06g46920 | dihydroflavonol-4-reductase |
| LOC_Os06g48180 | glycosyl hydrolases family 16 |
| LOC_Os06g48300 | protein phosphatase 2C |
| LOC_Os06g48310 | RNA recognition motif containing protein |
| LOC_Os06g48460 | retrotransposon protein, putative, Ty3-gypsy subclass |
| LOC_Os06g48500 | expressed protein |
| LOC_Os06g48960 | AIG2-like family domain containing protein |
| LOC_Os07g03180 | GCRP3 - Glycine and cysteine rich family protein precursor |
| LOC_Os07g04820 | protein kinase |

| | |
|---|---|
| LOC_Os07g06390 | expressed protein |
| LOC_Os07g06490 | DNA binding protein |
| LOC_Os07g10950 | expressed protein |
| LOC_Os07g15959 | expressed protein |
| LOC_Os07g24000 | AWPM-19-like membrane family protein |
| LOC_Os07g25900 | expressed protein |
| LOC_Os07g26110 | membrane associated DUF588 domain containing protein |
| LOC_Os07g28850 | retrotransposon protein, putative, unclassified |
| LOC_Os07g29760 | cysteine proteinase A494 precursor |
| LOC_Os07g32129 | expressed protein |
| LOC_Os07g32680 | retrotransposon protein, putative, unclassified |
| LOC_Os07g32920 | expressed protein |
| LOC_Os07g33700 | transposon protein, putative, unclassified |
| LOC_Os07g34690 | expressed protein |
| LOC_Os07g37730 | NADH-ubiquinone oxidoreductase, mitochondrial precursor |
| LOC_Os07g38130 | polygalacturonase inhibitor 1 precursor |
| LOC_Os07g41580 | histone-like transcription factor and archaeal histone |
| LOC_Os07g43950 | RNA recognition motif containing protein |
| LOC_Os07g44430 | peroxiredoxin |
| LOC_Os07g48630 | ethylene-insensitive 3 |
| LOC_Os08g01370 | expressed protein |
| LOC_Os08g01530 | expressed protein |
| LOC_Os08g02180 | expressed protein |
| LOC_Os08g02480 | retrotransposon protein, putative, unclassified |
| LOC_Os08g03220 | retrotransposon protein, putative, Ty3-gypsy subclass |
| LOC_Os08g07390 | mla1 |

| | |
|---|---|
| LOC_Os08g09110 | NB-ARC domain containing protein |
| LOC_Os08g09410 | OsFBX265 - F-box domain containing protein |
| LOC_Os08g12449 | expressed protein |
| LOC_Os08g13620 | retrotransposon protein, putative, Ty3-gypsy subclass |
| LOC_Os08g14195 | expressed protein |
| LOC_Os08g15050 | CCT/B-box zinc finger protein |
| LOC_Os08g19360 | retrotransposon protein, putative, unclassified |
| LOC_Os08g23070 | retrotransposon protein, putative, Ty3-gypsy subclass |
| LOC_Os08g23220 | expressed protein |
| LOC_Os08g23870 | late embryogenesis abundant group 1 |
| LOC_Os08g26840 | plant protein of unknown function domain containing protein |
| LOC_Os08g27170 | calmodulin binding protein |
| LOC_Os08g29240 | retrotransposon protein, putative, Ty3-gypsy subclass |
| LOC_Os08g29340 | ligA |
| LOC_Os08g30510 | expressed protein |
| LOC_Os08g32060 | spotted leaf 11 |
| LOC_Os08g32520 | expressed protein |
| LOC_Os08g34850 | retrotransposon protein, putative, unclassified |
| LOC_Os08g35160 | heat shock protein DnaJ |
| LOC_Os08g35820 | transposon protein, putative, CACTA, En/Spm sub-class |
| LOC_Os08g36910 | alpha-amylase precursor |
| LOC_Os08g36970 | expressed protein |
| LOC_Os08g37345 | DNA binding protein |
| LOC_Os08g38730 | expressed protein |
| LOC_Os08g40910 | expressed protein |
| LOC_Os08g42420 | expressed protein |

| | |
|---|---|
| LOC_Os09g02180 | expressed protein |
| LOC_Os09g07150 | expressed protein |
| LOC_Os09g09020 | retrotransposon protein, putative, unclassified |
| LOC_Os09g09040 | expressed protein |
| LOC_Os09g09210 | expressed protein |
| LOC_Os09g10470 | retrotransposon protein, putative, Ty3-gypsy subclass |
| LOC_Os09g12150 | OsFBX310 - F-box domain containing protein, expressed |
| LOC_Os09g12190 | transposon protein, putative, CACTA, En/Spm sub-class |
| LOC_Os09g15520 | oleosin |
| LOC_Os09g15670 | protein phosphatase 2C |
| LOC_Os09g20480 | transporter |
| LOC_Os09g21900 | expressed protein |
| LOC_Os09g26999 | keratin-associated protein 5-4 |
| LOC_Os09g27760 | retrotransposon protein, putative, Ty3-gypsy subclass |
| LOC_Os09g30418 | heat shock protein |
| LOC_Os09g34320 | expressed protein |
| LOC_Os09g36750 | L-ascorbate peroxidase 4 |
| LOC_Os09g39730 | Core histone H2A/H2B/H3/H4 domain containing protein |
| LOC_Os09g39850 | AP2 domain containing protein |
| LOC_Os10g08960 | expressed protein |
| LOC_Os10g10920 | retrotransposon protein, putative, Ty3-gypsy subclass |
| LOC_Os10g12330 | retrotransposon protein, putative, unclassified |
| LOC_Os10g20470 | MATE efflux family protein |
| LOC_Os10g20810 | transposon protein, putative, CACTA, En/Spm sub-class |
| LOC_Os10g28080 | glycosyl hydrolase |
| LOC_Os10g29230 | MBTB49 - Bric-a-Brac, Tramtrack, Broad Complex BTB domain with Meprin and TRAF Homology MATH |

| | |
|---|---|
| LOC_Os10g30150 | universal stress protein domain containing protein |
| LOC_Os10g32640 | retrotransposon protein, putative, Ty3-gypsy subclass |
| LOC_Os10g33010 | retrotransposon protein, putative, unclassified |
| LOC_Os10g35050 | aquaporin protein |
| LOC_Os10g35070 | alpha-galactosidase precursor |
| LOC_Os10g36180 | expressed protein |
| LOC_Os10g38110 | cytochrome P450 |
| LOC_Os10g38170 | expressed protein |
| LOC_Os10g38360 | glutathione S-transferase |
| LOC_Os10g38470 | glutathione S-transferase |
| LOC_Os10g40040 | expressed protein |
| LOC_Os10g41480 | phospho-2-dehydro-3-deoxyheptonate aldolase, chloroplast precursor |
| LOC_Os11g01530 | ferritin-1, chloroplast precursor |
| LOC_Os11g04409 | expressed protein |
| LOC_Os11g05614 | no apical meristem protein |
| LOC_Os11g05640 | bZIP transcription factor domain containing protein |
| LOC_Os11g07911 | expressed protein |
| LOC_Os11g09864 | wali7 |
| LOC_Os11g10630 | retrotransposon protein, putative, unclassified |
| LOC_Os11g13620 | expressed protein |
| LOC_Os11g13630 | gibberellin receptor GID1L2 |
| LOC_Os11g15690 | retrotransposon protein, putative, Ty3-gypsy subclass |
| LOC_Os11g26360 | retrotransposon protein, putative, Ty3-gypsy subclass |
| LOC_Os11g26570 | dehydrin |
| LOC_Os11g26760 | dehydrin |
| LOC_Os11g26780 | dehydrin |

| | |
|---|---|
| LOC_Os11g26790 | dehydrin |
| LOC_Os11g27520 | transposon protein, putative, CACTA, En/Spm sub-class |
| LOC_Os11g29570 | retrotransposon protein, putative, Ty3-gypsy subclass |
| LOC_Os11g30910 | sulfotransferase domain containing protein |
| LOC_Os11g32890 | expressed protein |
| LOC_Os11g33394 | plant protein of unknown function domain containing protein |
| LOC_Os11g34640 | expressed protein |
| LOC_Os11g36810 | expressed protein |
| LOC_Os11g37870 | stripe rust resistance protein Yr10 |
| LOC_Os11g41690 | retrotransposon protein, putative, unclassified |
| LOC_Os11g42490 | retrotransposon protein, putative, unclassified |
| LOC_Os11g43060 | transposon protein, putative, unclassified |
| LOC_Os11g43990 | expressed protein |
| LOC_Os11g44680 | calmodulin binding protein |
| LOC_Os11g47870 | GRAS family transcription factor domain containing protein |
| LOC_Os11g47910 | SCARECROW, putative, expressed |
| LOC_Os12g03770 | transposon protein, putative, CACTA, En/Spm sub-class |
| LOC_Os12g05210 | expressed protein |
| LOC_Os12g07440 | expressed protein |
| LOC_Os12g08020 | expressed protein |
| LOC_Os12g08700 | expressed protein |
| LOC_Os12g12140 | expressed protein |
| LOC_Os12g12180 | retrotransposon protein, putative, Ty1-copia subclass |
| LOC_Os12g14260 | retrotransposon protein, putative, Ty3-gypsy subclass |
| LOC_Os12g19140 | retrotransposon protein, putative, unclassified |
| LOC_Os12g24020 | rhodanese-like domain containing protein |

| | |
|---|---|
| LOC_Os12g24260 | transposon protein, putative, CACTA, En/Spm sub-class |
| LOC_Os12g26020 | expressed protein |
| LOC_Os12g26340 | retrotransposon protein, putative, Ty3-gypsy subclass |
| LOC_Os12g28570 | transposon protein, putative, CACTA, En/Spm sub-class |
| LOC_Os12g28590 | ATPase 2 |
| LOC_Os12g29190 | retrotransposon protein, putative, Ty3-gypsy subclass |
| LOC_Os12g29400 | GRAM domain containing protein |
| LOC_Os12g30214 | retrotransposon protein, putative, Ty3-gypsy subclass |
| LOC_Os12g36630 | universal stress protein domain containing protein |
| LOC_Os12g36850 | pathogenesis-related Bet v I family protein |
| LOC_Os12g36880 | pathogenesis-related Bet v I family protein |
| LOC_Os12g37519 | retrotransposon protein, putative, unclassified |
| LOC_Os12g40020 | retrotransposon protein, putative, unclassified |
| LOC_Os12g43640 | receptor-like protein kinase HAIKU2 precursor |
| LOC_Os12g43870 | expressed protein |
| LOC_Os12g44180 | nodulin |
