# Supplementary material for: Non-canonical regulation of SPL transcription factors by a human OTUB1-like deubiquitinase defines a new plant type rice associated with higher grain yield
Source: Cell Res. 2017 Aug 4;27(9):1142–56. doi: 10.1038/cr.2017.98 (PMC5587855; doi:10.1038/cr.2017.98)
Supplement: Supplementary information, Table S3 — The primer sequences used for map-based cloning and genotyping assays. [file cr201798x10.pdf]

Supplementary information, Table S3.

The primer sequences used for map-based cloning and genotyping assays.

Primers	Chr.	Forward (5’- 3’)	Reverse (5’- 3’)
RM1282	1	aagcatgacagctgcaagac	ggggatgaagggtaatttcg
RM3251	1	ccttcgagtgcatgtccc	gaacacgaggagaatgagcc
RM8111	1	aggtaactaagctaggtgtt	taggtacagtaataccaagc
RM1287	1	ggaagcatcatgcaatagcc	ggccgtagttttgctactgc
STS-1-72.8	1	atgctctgtttggcttatttcacg	atacagttcgtccgctacaggc
STS-1-103	1	catctcaaagtcgtgagtgcaac	atacgtgacccctccgagct
RM8097	1	tacatacacgttcatgtgcc	cgagcgtaggaagactacc
RM3738	1	gaaaggtgatgggagagcag	tgacaccctctctccacctc
STS-1-170.4	1	gtgcatcgaactcgtacaacc	tctagtgttgggggtcacctc
RM1347	2	aacaaattaaactgccaag	gtcttatcatcagaactgga
STS-2-55.4	2	tgactatgtaaggttgctgctg	acagctccacagcaaagaacc
STS-2-89.9	2	atcctgatctctcctgtgcc	agatgtaaacccaataggacc
RM3763	2	tctctgaacacacccacacc	tgttttgatctcagctcccc
STS-2-144.7	2	aggatgcaggtgtgatgatac	tcaaagccaaattggactcc
STS-2-157.9	2	tcgttgttggagagtttgtgcc	agatacgttaaacgatgtg
STS-3-17.9	3	ttggagagacgagcgagagag	agtgttggtgagcatagcagttg
STS-3-36.9	3	tggccgttgaggcgattag	cagagtcttactgctaaccccgt
STS-3-57.2	3	gcgtcgtaatgtaatggctgg	ttgtttgacctcacacactctgc
RM6676	3	tgcataacacccaaatgccttc	tatgttacaatgttcacggtcc
RM3513	3	acgctgtggctatgcctttgg	acatggccttgtagtagacgagag
RM1350	3	atcagcaagaaagctctgctcc	aggaaattcgccctagtagatag
RM7000	3	tgaactcgttcttttgcaccg	acgaagtccccttcttttcaac
STS-4-3.1	4	agaatagagtgcatcatcgtc	aacctgataggtggaagatgtac
RM307	4	gtactaccgacctaccgttcac	ctgctatgcatgaactgctc
RM1359	4	ccaaaggtcaacgaattcta	cggctggttaattaatcaaa

RM252	4	ttcgctgacgtgataggttg	atgacttgatcccgagaacg
RM303	4	gcatggccaaatattaaagg	ggttggaaatagaagttcggt
RM1113	4	gggcgcatgtgtatttcttc	tggggaaaaaccacaagcc
RM17713	5	ttgtaaccaccagcagcaggg	agcaatggtacaaatagccaagc
RM13	5	tccaacatggcaagagagag	tatcacattcgattccagcatg
RM18384	5	tacgtaccaacaggcggagg	ttcttggtgctatcttcgtgctc
RM1237	5	ctccgcgagctttagaagag	cacatactctggctctcccg
RM18751	5	atcccgtgtgttggcttagaatc	tccaaacatcagaaagcaagcac
RM3476	5	ttaccacaaggattctcgtcg	tccacggttaagataaatgcatc
RM587	6	acgcgaacaaattaacagcc	ctttgctaccagtagatccagc
RM276	6	tagcaagacatggacctcaacg	tcctccatcgagcagtatcag
RM6836	6	ttgttgtatacctcatcgac	agggtaagacgtttaacttg
RM7434	6	ggaggaaaggttggagaagg	tttcccgtattccatgagcc
RM340	6	tagcagcaaccctattctggag	ataagtcatgatggtaaacctgtc
RM412	6	cacttgagaaagttagtgcagc	cccaaacacacccaaatac
RM427	7	tcactagctctgccctgacc	tgatgagagttggttgcgag
RM1186	7	aataatctgagccagctgcg	ctgcgggtaggcagctatac
RM5481	7	tgttatgtgagccataatga	ctttcttaatgaagctccaa
RM455	7	aacaacccaccacctgtctc	agaaggaaaagggctcgatc
RM234	7	acagtatccaaggccctgg	cacgtgagacaaagacggag
RM248	7	tccttgtgaaatctggtccc	gtagcctagcatggtgcatg
RM407	8	gattgaggagacgagccatc	ctttttcagatctgcgctcc
P154	8	cattgggtttgtgcattcag	caacgacccatattccaacc
P155	8	ggggtgacttgaggtagtgg	gcgacttcagttctccacct
RM531	8	gaaacatcccatgttcccac	tcggtttttcagactcggtc
P394	8	tgttggatttgttctttaccgc	cacaacaagctcgacgtgatc
RM284	8	atctctgatactccatccatcc	cctgtacgttgatccgaagc
RM556	8	actccaaacctcactgcacc	tagcacactgaacagctggc
RM80	8	ttgaaggcgctgaaggag	catcaacctcgtcttcaccg

P6049	8	cgtgttaaggcatccacaat	ccaaacaccccattagttactt
P3888	8	gatggatggagtactttatatcagc	gtcccggttattttaacctagac
P712	8	ttcagattgggtatgctcat	cattcagactttcagaggca
P5528	8	attgaaccaagccagctcac	tggtcgatattttaaaagtttgcc
P4464	8	ctgaatttaaccagttttttggg	ccatcctgagttaattagcaattg
P135	8	cctcctcctcctccagcaac	ccgccatctctcgctctctc
P139	8	ttggaggcatgattaggata	tcagtggatttgcagacgtt
P143	8	aatgattagccttgaagattggtcc	ggaaggaggttgcaataatttg
P168	8	aattgcgtgcgtcagcaatg	ggcatagctccactccaaaagcta
P3948	8	tggaaggtactacggcatattg	cagagcacgaaaccaaccat
P3914	8	tgccggagagcaccactgc	tctccgccggattggcctt
P4632	8	acaatgcaattacactgtagctg	tcccttcagttctcattcaaa
P4704	8	ggtgattagcagcggtcaaa	ggtatctgtcctcttcgtgtcg
P351	8	ccgaaagcgaaaattctga	gaagggaagatcgagttgaca
RM444	9	gctccacctgcttaagcatc	tgaagaccatgttctgcagg
RM3700	9	aaatgccccatgcacaac	ttgtcagattgtcaccaggg
RM242	9	ggccaacgtgtgtatgtctc	tatatgccaagacggatggg
RM7048	9	caacccctaatttcacgctc	gacttcactggcactggatg
RM474	10	aagatgtacgggtggcattc	tatgagctggtgagcaatgg
RM1873	10	ctgacaggacattaaaaaac	cctcatccttaatctcttta
RM271	10	tcagatctacaattccatcc	tcggtgagacctagagagcc
RM304	10	tcaaaccggcacatataagac	gatagggagctgaaggagatg
RM228	10	ctggccattagtccttgg	gcttgcggctctgcttac
RM496	10	gacatgcgaacaacgacatc	gctgcggcgctgttatac
RM7577	11	gtgtactgccatgaaaggcc	gaagtgcctttgcaggagag
RM552	11	cgcagttgtggatttcagtg	tgctcaacgtttgactgtcc
RM3428	11	attcatgcttcctttcagtg	gattactggtttgccatttg
RM287	11	ttccctgttaagagagaaatc	gtgtatttggtgaaagcaac
RM2191	11	aataagggagagccaatctg	tagtagatggccgttctctc

RM144	11	tgccctggcgcaaatttgatcc	gctagaggagatcagatggtagtgcatg
RM20A	12	atcttgtccctgcaggtcat	gaaacagaggcacatttcattg
RM19	12	caaaaacagagcagatgac	ctcaagatggacgccaaga
RM3472	12	atcgcaagaactccgtgaag	cgcttttgagctcgcctc
RM7119	12	aggctgaggcttataggcag	ggatgatacaacttgacccc
RM1246	12	ctcgatcccctagctctc	tcacctcgttctcgatcc
RM1103	12	cagctgctgctactacaccg	ctactccacgtccatgcatg
RM17	12	tgccctgttattttcttctctc	ggtgatcctttcccatttca
