# Supplementary material for: Non-canonical regulation of SPL transcription factors by a human OTUB1-like deubiquitinase defines a new plant type rice associated with higher grain yield
Source: Cell Res. 2017 Aug 4;27(9):1142–56. doi: 10.1038/cr.2017.98 (PMC5587855; doi:10.1038/cr.2017.98)
Supplement: Supplementary information, Table S4 — The primer sequences used for DNA constructs and transcript analysis. [file cr201798x11.pdf]

Supplementary information, Table S4.

The primer sequences used for DNA constructs and transcript analysis.

Primers	Forward (5'- 3')	Reverse (5'- 3')
pOsOTUB1.1	gaattcgagttgaagttgttgctgctgtca	ggtacccgagagctcgaccgacacg
gOsOTUB1.1	cccgggatgggcggggactactaccact	gtcgactcacttcggggtagagaatgtcg
pOsOTUB1.2	gaattcgccgctgcgcccatatgacc	ggtacccgataaaagcagtttccatc
gOsOTUB1.2	cccgggatgttttcctacttggtattattt	gtcgactcacttcggggtagagaatgtcg
OsOTUB1.1-OE	cccgggatgggcggggactactaccact	gtcgactcacttcgggtagagaatgtcg
OsOTUB1.2-OE	cccgggatgttttcctacttggaacatatc	gtcgactcacttcgggtagagaatgtcg
OsOTUB1.1-GFP	cccgggatgggcggggactactaccact	gtcgaccttcgggtagagaatgtcgtag
OsOTUB1.2-GFP	cccgggatgttttcctacttggaacatatc	gtcgaccttcgggtagagaatgtcgtag
CIRSPR -OsOTUB1	cagctggaaagtgttctgcgttttagagctagaaat	cagaacactttccagctgacggcagccaagccagca
OsUBC13-OE	gtcgacatggccaacagcaacctcccccgg	ctgcagttatgcaccgctggcatacaggcg
RNAi-OsUBC13-L	actagtcccggcgaatcatcaaggagac	agatctgaactgtccgaatctgaagggc
RNAi- OsUBC13-R	tctagacccggcgaatcatcaaggagac	ggatccgaactgtccgaatctgaagggc
OsSPL14-OE	gaattcctatggagatggccagtggaggag	ggatcccctacagagaccaatccatcgtgtt
RNAi-OsSPL14-L	actagtaagaacaaggggaagggcgtg	agatctaaaggggtttgcggcctcct
RNAi-OsSPL14-R	tctagaaagaacaaggggaagggcgtg	ggatccaaaggggtttgcggcctcct
BD-OsOTUB1.1	ggatccatgggcggggactactaccact	gaattctcacttcgggtagagaatgtcg
BD-OsOTUBΔC	ggatccatgttggaacatatcctagagac	gaattctcacttcgggtagagaatgtcg
AD-OsUBC13	ggatccatggccaacagcaacctcccccgg	gtcgactgcaccgctggcatacaggcg
BD-OsSPL14ΔN2	gaattcatgccgccgcggtgccaggtgga	ggatcccctacagagaccaatccatcgtgtt
cYFP-OsOTUB1.1	gtcgacaatgggcggggactactaccact	ggatcctcacttcgggtagagaatgtcg
nYFP-OsUBC13	gtcgacaatggccaacagcaacctcccccgg	ggatccttatgcaccgctggcatacaggcg
nYFP-OsSPL14	gagctcatggagatggccagtggaggag	ggatccctacagagaccaatccatcgtgtt
nYFP-OsSPL14ΔN1	gagctcatggcaggcggcggcggcactgg	ggatccctacagagaccaatccatcgtgtt
nYFP-OsSPL14ΔN2	gagctcatgccgccgcggtgccaggtgga	ggatccctacagagaccaatccatcgtgtt

nYFP-OsSPL14ΔN3	gagctcatgagctttacgttggatttctc	ggatccctacagagaccaatccatcgtgtt
nYFP-OsSPL14ΔN4	gagctcatgtgggatactactacccacagt	ggatccctacagagaccaatccatcgtgtt
nYFP-OsSPL14ΔC1	gagctcatggagatggccagtggaggag	ggatccctaccatggctgggttgacagaa
nYFP-OsSPL14ΔC2	gagctcatggagatggccagtggaggag	ggatccctatctgaacctgcgatgctcac
nYFP-OsSPL14ΔC3	gagctcatggagatggccagtggaggag	ggatccctacggcggcggcggcggcggcg
nYFP-OsSPL14ΔC4	gagctcatggagatggccagtggaggag	ggatccctatgccgcggcggcgtcctcga
nYFP-SBP	gagctcatgccgccgcggtgccaggtgga	ggatccctaggtttgcggcctcctccggc
YFP-SPL14ΔSBP-1	gagctcatggagatggccagtggaggag	gcgtgatgccaaaggggtttgcacgcccttccccttgttct
YFP-SPL14ΔSBP-2	caaaccccttggcatcacgc	ggatccctacagagaccaatccatcgtgtt
OsSPL1	cgagctcatgtcgagtgggctcaagaa	cgggatcctcacttggggcctgaacgca
OsSPL2	gcgtcgacaatggattgggacgccaagat	cgggatccctaccacgatgagaaaggaa
OsSPL3	gcgtcgacaatgggttcttttgggatgga	cgggatccctataatgcaaatagaatct
OsSPL4	gcgtcgacaatggattggatgcctcctcc	cgggatccttaatgaaatgacatgcagc
OsSPL5	cgagctcatggcggtgccagcggcggc	cgggatccctagatgaaatccacctcga
OsSPL6	gcgtcgacaatggaggctgcccgggtcgg	cgggatcctcacattggtccacgttcta
OsSPL7	gcgtcgacaatggaaggaaacggctgcggc	cgggatcctcagaccacgcgggcgccct
OsSPL8	gcgtcgacaatgatgaacgttccatccgc	cgggatccctagtgatcgaagtcgagat
OsSPL9	gcgtcgacaatggacgcccccggcggcggcg	cgggatccctatgatgagtagttcctag
OsSPL10	ggactagtatgatgagcggtaggatgaa	cgggatccctacatgaagtcgacctcga
OsSPL11	gcgtcgacaatggagtgcaaccccgtctc	cgggatcctcaatgtatctggttcagac
OsSPL12	gcgtcgacaatggcttcttttgggatgaa	ggactagttcagtgcagatggccatagc
OsSPL13	cgagctcatggaccgcaaggacaaggc	cgggatccttatctgatctggaacggcg
OsSPL14	cattgggtttgtgcattcag	caacgacccatattccaacc
OsSPL15	gcgtcgacaatgcagagggaagtggggcc	cgggatccttatatcgtaccaaaatcca
OsSPL16	gtcgacaatggagtgggatctcaagat	ggatccctactgccatgagaacggca
OsSPL17	gcgtcgacaatggcgaccggcggcagcgg	cgggatccctacagagaccagttcatgg
OsSPL18	cgagctcatggattgggatctcaagat	cgggatccctactgccacgagaatggga
OsSPL19	cgagctcatggagtgggcggcggcggc	cgggatccctacacctgccaagagaat
OsOTUB1	gattgaggagacgagccatc	ctttttcagatctgcgctcc

OsUBC13	ggggtgacttgaggtagtgg	gcgacttcagttctccacct
OsTB1	gaaccactcatcgtccacca	ctgatcctgctgatgctgct
DEP1	gcgagatcacgttcctcaag	tgcagtttggcttacagcat
OsRCN2	caaccccacggtgaagatga	acctgtggatgccgatgttt
OsMADS34	gagatcgacgtagaggcagc	taggccatccactcaggagg
Os11g47870	agctgcacatcgtggactac	ttgcagcaatggcttggaac
Os06g38294	tcgttcttgtggtggaggtg	aggtagatggcgtaggtggt
Actin1	ccactatgttccctggcatt	gtactcagccttggcaatcc
DEP1-GTAC-motif	aatttattcccttgctgtttcatttcgtacgtactccgc	gctatggccgcatcccgagcgcggagtacgtacgaaatg
	gctcgggatgcggccatagc	aaacagcaagggaataaatt
